# Evidence of selection on splicing-associated loci in human populations and relevance to disease loci mapping

**DOI:** 10.1038/s41598-017-05744-9

**Published:** 2017-07-20

**Authors:** Eric R. Gamazon, Anuar Konkashbaev, Eske M. Derks, Nancy J. Cox, Younghee Lee

**Affiliations:** 1 Division of Genetic Medicine, Department of Medicine, Vanderbilt University, Nashville, TN 37235 USA; 2 Academic Medical Center, Department of Psychiatry and Department of Clinical Epidemiology, Biostatistics and Bioinformatics, University of Amsterdam, Amsterdam, The Netherlands; 3 Department of Biomedical Informatics, School of Medicine, University of Utah, Salt Lake City, UT 84108 USA; 4 Section of Genetic Medicine, Department of Medicine, The University of Chicago, Chicago, IL 60637 USA; 5 Translational Neurogenomics Group, QIMR Berghofer, Brisbane, QLD 4006 Australia

## Abstract

We performed a whole-genome scan of genetic variants in splicing regulatory elements (SREs) and evaluated the extent to which natural selection has shaped extant patterns of variation in SREs. We investigated the degree of differentiation of single nucleotide polymorphisms (SNPs) in SREs among human populations and applied long-range haplotype- and multilocus allelic differentiation-based methods to detect selection signatures. We describe an approach, sampling a large number of loci across the genome from functional classes and using the consensus from multiple tests, for identifying candidates for selection signals. SRE SNPs in various SNP functional classes show different patterns of population differentiation compared with their non-SRE counterparts. Intronic regions display a greater enrichment for extreme population differentiation among the potentially tissue-dependent transcript ratio quantitative trait loci (trQTLs) than SRE SNPs in general and includ outlier trQTLs for cross-population composite likelihood ratio, suggesting that incorporation of context annotation for regulatory variation may lead to improved detection of signature of selection on these loci. The proportion of extremely rare SNPs disrupting SREs is significantly higher in European than in African samples. The approach developed here will be broadly useful for studies of function and disease-associated variation in the human genome.

## Introduction

Alternative splicing (AS) increases human proteomic diversity by enabling multiple, distinct transcripts to be generated from the same precursor gene^1^. In human cells, nearly 90% of protein-coding genes may generate multiple transcript isoforms^2^. As a molecular process, splicing is performed by the spliceosome, a macromolecule (consisting of small nuclear ribonucleoproteins) involved in the recognition of exon-intron boundaries and in the catalysis of the reactions that splice introns and join exons^3^. The exquisite process depends on how precisely the spliceosome recognizes the exon-intron boundary with consensus sequence-based guide such as the branch point sequence and polypyrimidine tract. Non-splice site motifs involved in the regulation of splicing are known as splicing regulatory elements (SREs), which are hexameric (i.e., six base pairs in length) sequences classified (based on location and effect on splicing) as intronic splicing enhancers (ISEs), intronic splicing silencers (ISSs), exonic splicing enhancers (ESEs), and exonic splicing silencers (ESSs)^4, 5^. SREs are cis-acting elements and exert their regulatory function via recruitment of sequence-dependent RNA-binding factors, to activate or repress adjacent splice sites. For instance, most ESEs recruit members of the serine/arginine-rich (SR)^6^ protein family whereas ESSs are typically bound by repressor proteins of the hnRNP class. Thus, the splicing process is a complex sequence-mediated interaction between the spliceosome (trans-acting factors) and the pre-mRNA (cis-acting elements). A single change at any position within an SRE may turn off its regulatory function and disrupt the binding accuracy of the spliceosome to exon-intron boundaries, possibly generating a defective, disease-causing protein. Indeed, disruptions of normal splicing patterns are implicated in a variety of human diseases^7–14^. It has been estimated that as high as 15% of disease-causing mutations affect splicing^9, 15–17^. Thus, sequence variation in SREs, by disrupting the splicing machinery, may play a role in human phenotypic diversity.

We have previously shown that sequence variations in ISEs are enriched among genetic variants that have been identified by genome-wide association studies (GWAS) to be reproducibly associated with complex human traits, including a broad spectrum of common diseases and quantitative traits^18^. However, the contribution of AS to disparities in disease risk remains to be fully characterized although its significance as a mechanism for conferring disease risk in diverse populations is increasingly being recognized^19^ such as through recent experimental evidences in critical oncogenes (i.e. *BCLXL, MET, RASGPR2, PI3K, and MDM2* ^20–25^).

Splicing differences between individuals are common in human populations^21^. In a comparison of transcript levels obtained from lymphoblastoid cells derived from individuals of European and African descent, ~10% of the investigated genes showed population-specific splicing ratios^24^. In prostate cancer, transcript isoforms expressed in African Americans translate into more aggressive forms of oncogenes^19^. Splicing-associated variants in the insulin gene that are more common or unique in individuals of African descent raised the hypothesis of the influence of selection resulting from the transition of an out-of-Africa ancestral population to primitive agriculture^26^. To investigate the genetic basis underlying differences in splicing in human populations, we performed comprehensive analyses of genetic variants in SREs, including the degree of population differentiation in SRE variants among continental populations using whole-genome sequence data, and of the extent to which the observed patterns of differentiation at these genomic loci are consistent with the action of selection using long-range haplotype- and multilocus allelic differentiation- based methods.

## Results

Our primary aim is to test whether variants affecting splicing would show greater population differentiation in allele frequency and evidence for selection than matched variants not affecting splicing. Towards this end, we quantified the degree of population differentiation using *F*_ST_^27^ (see Methods) for the 1000 Genomes Project (TGP, phase 3) SNPs. Population differentiation as a test for selection is, however, sensitive to demographic history (e.g., migration) and the *F*_ST_ statistic can show wide variation even at loci under neutrality^28, 29^. Hence, within each broad functional class (see Methods), we compared outlier splicing-associated loci with the empirical distribution of population differentiation across the genome. Furthermore, selection signatures derived from local scans for reduced variation may be confounded by demographic processes (e.g., population bottleneck or recent founder effects). We therefore applied several alternative methods for detecting selection, including approaches based on cross-population multi-locus allelic differentiation and on cross-population extended haplotype homozygosity^30, 31^, to identify candidate SREs with multiple signatures of selection.

**Figure 1:**
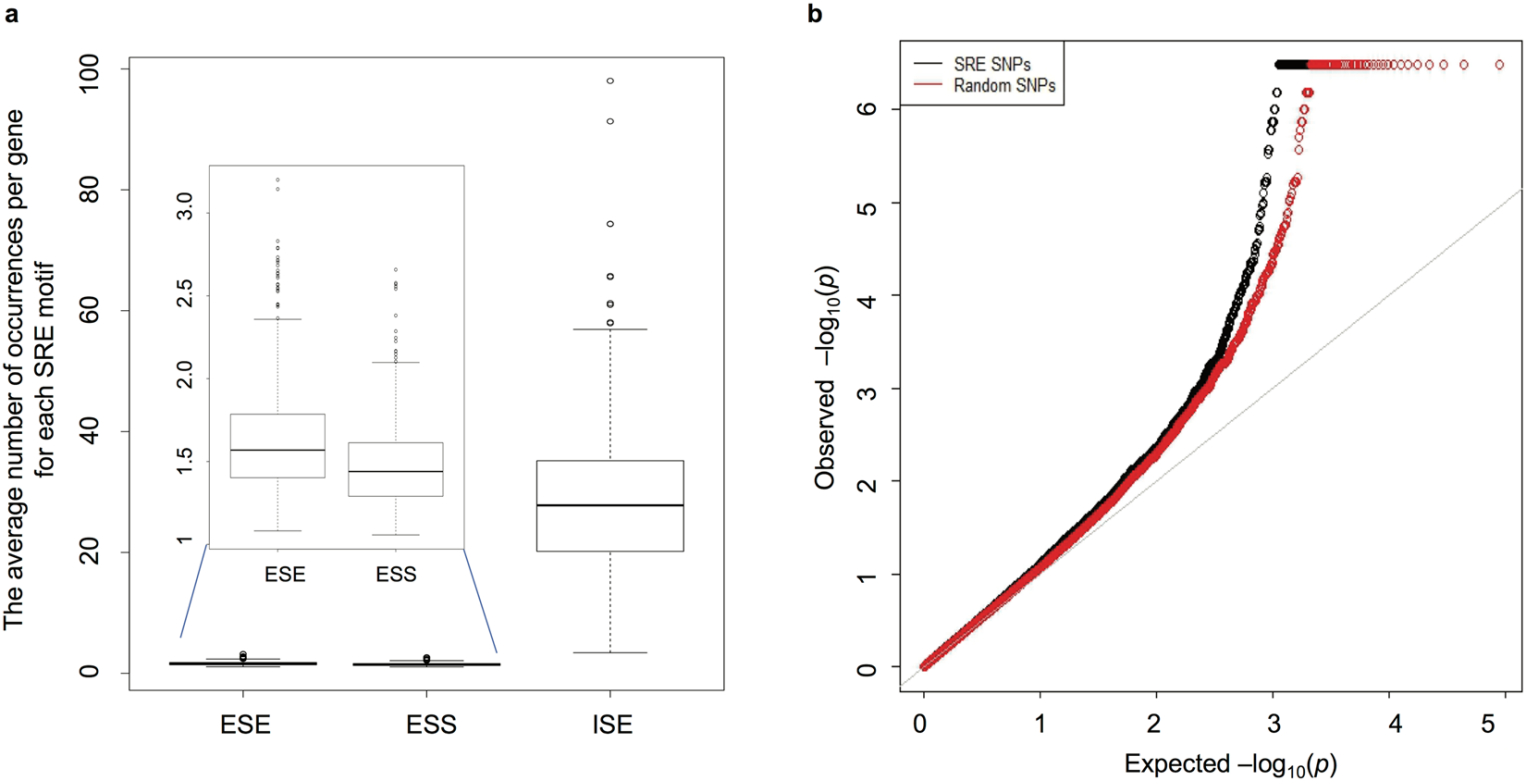
SRE SNPs: distribution of SRE motifs and evidence of impact on splicing. (**a**) The box plot shows the distribution of the number of occurrences per gene for each class of SRE motifs. (**b**) The Q-Q plot shows the distribution of p-values from the association with splicing ratios of genes (estimated using sQTLseekeR applied to GTEx first-phase data) in whole blood for the set of SRE SNPs and for a random set of SNPs matched on relevant SNP attributes. The leftward shift in the Q-Q plot was observed for the SRE SNPs relative to all such random sets (n = 1000).

A SNP was annotated as an SRE SNP if it is within an SRE site (a hexameric splicing motif) and is located immediately adjacent to the skipped exon or the exon embedding the candidate SNP is skipped (see Methods)^32^. An SRE SNP was also functionally classified into the following SNP classes: intronic, synonymous, non-synonymous, and loss-of-function. See Supplementary Table 1 for the number of SRE and non-SRE SNPs by SNP functional category included in our analyses. We utilized 979, 496, and 432 hexameric motifs derived from a neighborhood inference algorithm to define ESEs, ESSs, and ISEs, respectively (see Methods); we found these motifs to be distributed across 19,844, 19,816, and 17,571 genes, respectively. The average number of SRE occurrences per gene is 1.62, 1.49, and 29.4 for ESEs, ESSs, and ISEs, respectively (Fig. 1a). The standard deviation for the number of instances per gene is 0.33, 0.26, and 13.56 for ESEs, ESSs, and ISEs, respectively. Furthermore, the ISE SNPs constitute nearly 11% of all intronic SNPs tested here. For SRE SNPs, the average minor allele frequency in AFR is 0.045 (std dev = 0.096) whereas the corresponding value in EUR is 0.035 (std dev = 0.094); the difference between the two groups is significant (Mann-Whitney U test P < 2.2 × 10^−16^). EUR (57%) has 1.7 times as many extremely rare variants (MAF < 0.001) disrupting SRE motifs as AFR (33%). This proportion of extremely rare SRE-disrupting variants is markedly higher than the proportion of non-synonymous SNPs (55.4% and 47.0% for European and African samples, respectively) and higher than the proportion of SNPs inferred to be “probably damaging” (15.9% versus 12.1% for European and African samples, respectively) for SNPs segregating in only one population from an early study^33^.

We sought additional support from RNA-Seq data for the role of the SRE SNPs on splicing. We utilized information on splicing QTLs (sQTLs) from the first-phase GTEx data in 9 tissues. The SRE SNPs are significantly enriched (empirical p-value < 0.001, n = 1000 random sets) for the best sQTLs per exon-exon link identified using Altrans^34, 35^ after matching on minor allele frequency (MAF), distance to intron/exon boundary, gene size, and extent of LD (see Methods). Furthermore, the SRE SNPs show a shift towards low p-values for the SNP associations with changes in the splicing ratios of genes (quantified using sQTLseekeR^36^) in whole blood compared to a random set (n = 1000) of SNPs matched on the same set of features (Fig. 1b).

### SRE SNPs in various SNP classes and comparison with non-SRE SNPs

For illustration and in downstream analyses, we focused on the AFR and EUR comparison unless otherwise stated.

**Figure 2:**
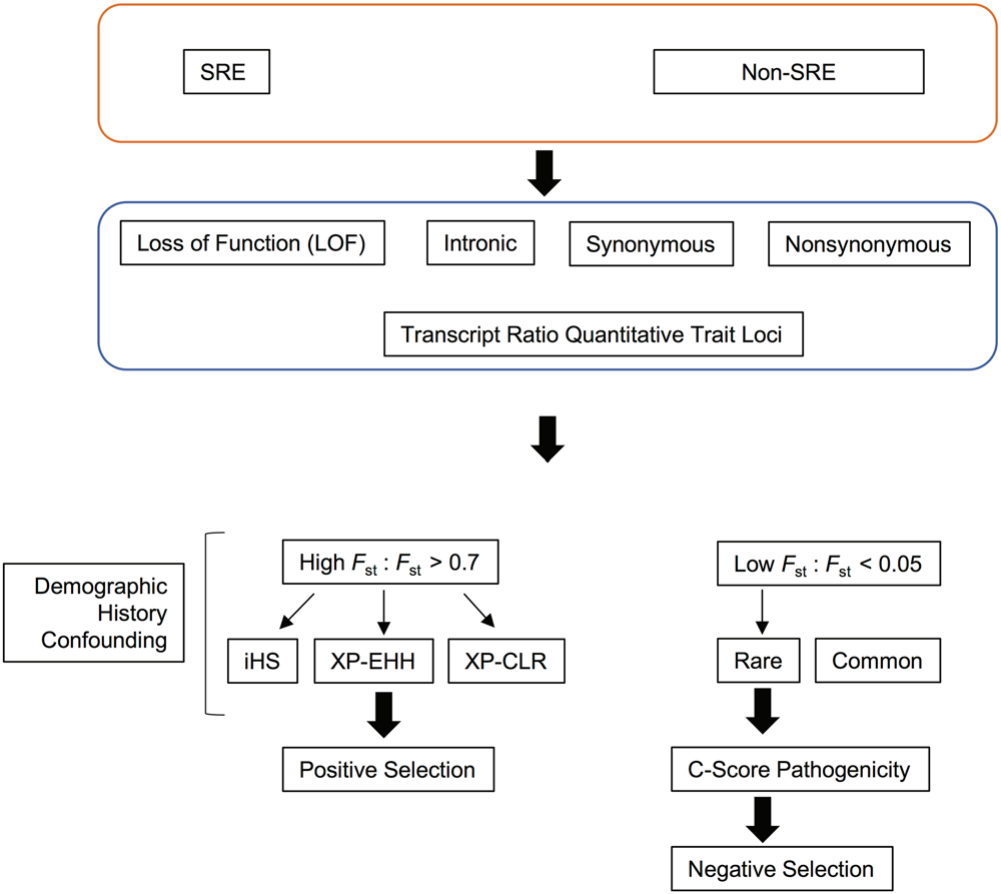
Illustration of overall analytic approach. In our analysis, SRE SNPs and non-SRE SNPs were classified according to four functional categories: LOF, intronic, synonymous and non-synonymous. We also considered the overlap of SRE SNPs with transcript ratio quantitative trait loci (GEUVADIS) and splicing QTLs (GTEx) identified in RNA-Seq studies in LCLs and in human tissues, respectively. We assessed the evidence for high population differentiation and complemented this with evidence for positive selection using (given the potential for confounding due to demographic history) one of 3 additional signatures (iHS, XP-EHH, and XP-CLR). We also evaluated the evidence for negative selection and incorporated a metric of pathogenicity.

Because demographic forces tend to impact loci genome-wide while selection tends to be more locus-specific, comparisons of specific SNP classes across the entire genome and the use of consensus calls from multiple signatures at candidate loci may facilitate detection of the effect of selection^37^. We thus sought to gain insights into selection on genetic variation affecting splicing by considering differences in population differentiation between SNP classes among the SRE SNPs as well as between SRE and non-SRE SNPs and sought additional support from haplotype-based and multi-locus methods for detecting signatures of selection (Fig. 2).

Negative selection (through its action on deleterious mutations) or balancing selection (by maintaining allelic variation between populations) acts to reduce *F*_ST_ while positive selection (via local geographic adaptation) increases *F*_ST_^37, 38^. Supplementary Figure 1 illustrates the global *F*_ST_ distribution for the SRE SNPs in the different SNP classes, showing differences between SNP classes in the proportion of low *F*_ST_ variants. LOF and non-synonymous SRE SNPs have a higher proportion of low *F*_ST_ variants than intronic and synonymous SRE SNPs (Supplementary Figure 2 for the ASN-EUR comparison and the AFR- ASN comparison, which shows a similar pattern). While enrichment for low *F*_ST_ has been previously reported for non-synonymous SNPs (and recapitulated here), we observed a substantially greater level of excess (i.e., more than two-fold in terms of percentage) of low population differentiation among the amino-acid altering SRE variants than among all amino-acid altering SNPs^39^. Furthermore, among the LOF and non-synonymous SRE SNPs with low *F*_ST_, we observed an excess of low derived-allele frequency variants (<0.05 in EUR, Supplementary Figure 3), consistent with negative selection, and no excess in the higher allele frequency bins, as one might find under balancing selection.

**Figure 3:**
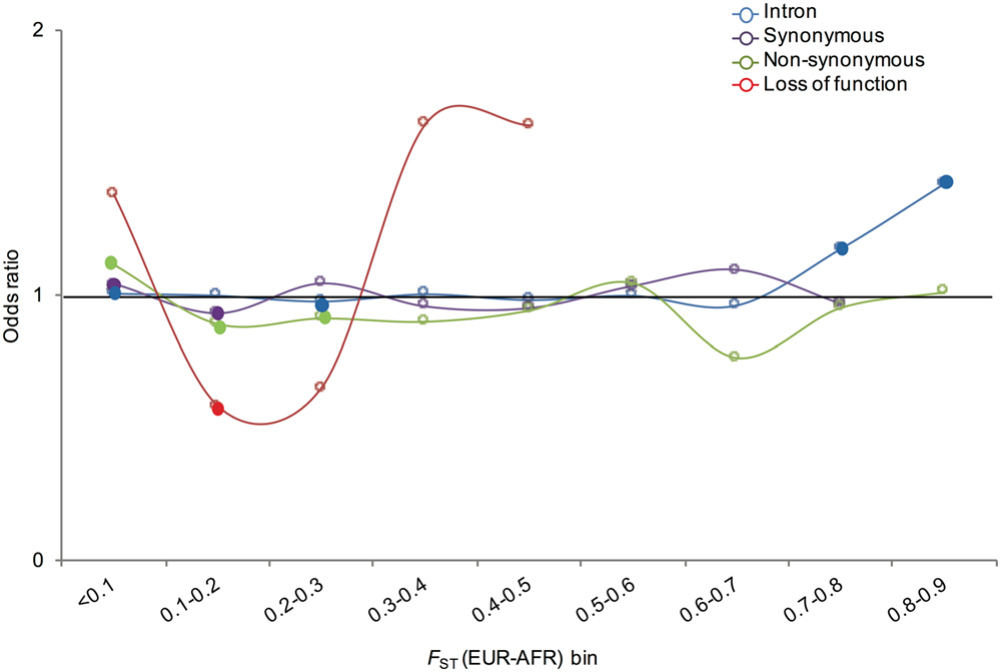
Relative proportion of SRE SNPs as a function of level of population differentiation (*F*_ST_). X-axis is *F*_ST_ bin from the AFR-EUR comparison. Y-axis represents the odds ratio (OR) of SRE SNPs to non-SRE SNPs in each SNP class. The non-SRE SNPs served as control in this comparison within each *F*_ST_ bin. The solid circle indicates statistically significant comparisons (P < 0.05). See Supplementary Figure 4 for the ASN-EUR comparison and the AFR-ASN comparison.

We then tested whether there is a significant difference in *F*_ST_ between SRE SNPs and non-SRE SNPs. We considered the proportion of SRE SNPs relative to the proportion of non-SRE SNPs among the different SNP categories as a function of *F*_ST_. Figure 3 illustrates this “odds ratio” (see Methods) in bins of *F*_ST_ in the AFR-EUR comparison (see Supplementary Figure 4 for the ASN-EUR comparison and the AFR-ASN comparison), showing that SRE SNPs have higher *F*_ST_ value than non-SRE SNPs. Note that the non-SRE SNPs across the genome served as a control in this comparison across the *F*_ST_ bins, which could enable detection of the effect of selection by identifying outlier SRE variants with respect to a genome-wide distribution.

In the next three sections, we “zoom in” on the evidence for extreme population differentiation and selection for variants in SREs within the SNP functional classes, allowing us to explore selection on potential regulatory loci independently of selective effects on the particular coding class.

### Selection on splicing motifs in intronic regions

We observed that intronic SRE SNPs show greater enrichment in the higher *F*_ST_ bins than intronic non-SRE SNPs, (Fig. 3, P = 2 × 10^−4^ at 0.7 < *F*_ST_ ≤ 0.8 and P = 6 × 10^−4^ at 0.8 < *F*_ST_ ≤ 0.9). Furthermore, among the intronic SRE SNPs, there is an enrichment (significant at P = 2.05 × 10^−11^ given the large number of SNPs in the bin) for low *F*_ST_ relative to intronic non-SRE SNPs.

The observed excess of extreme population differentiation (*F*_ST_ > 0.70) for the intronic SNPs in SRE is consistent with positive selection acting on these splicing motifs, but it may also result from genetic drift. To address this, we sought additional support from genome-wide scans of positive selection that utilize haplotype-based tests, namely, the integrated Haplotype Score (iHS)^31^ and the Cross Population Extended Haplotype Homozygosity (XP-EHH)^30, 40^. The iHS quantifies the extent of extended haplotype homozygosity at a SNP along the ancestral allele relative to the derived allele. The XP-EHH test is a cross-population approach to detecting high-frequency selective sweeps. Both scores were standardized (μ = 0, *σ* ^2^ = 1) for identifying outliers. Furthermore, we utilized the Cross Population Composite Likelihood Ratio (XP-CLR)^41^ test, which is based on the multilocus allele frequency differentiation between two populations.

We note that these methods detect selection within the timescale of ~25,000 years. A recently published approach, the Singleton Density Score (SDS)^42^, detects very recent changes in allele frequencies in a timescale (about 75 generations for samples of 3000 individuals) that is of an order of magnitude shorter than for these signatures (e.g., iHS detects a signal over > 1000 generations). Importantly, the approach enables detection of polygenic selection acting on a large number of loci across the genome. However, we find no significant difference in SDS score between the complete set of SNPs across the genome and SRE SNPs (Mann-Whitney U test P = 0.67) or the intronic SRE SNPs (Mann-Whitney U test P = 0.14).

An intronic SRE (ISE) SNP, rs2675347, located in the gene *SLC24A5*, known to be associated with natural skin control variation and previously shown to be under positive selection by genome-wide scans^40^, shows extreme population differentiation (*F*_ST_ = 0.71), XP-EHH (=3) and XP-CLR (=146). Furthermore, *SLC24A5* produces a transcript isoform in which the second exon is skipped (i.e., NM_205850 and ENST00000449382) and the SRE (ISE) SNP, rs2675347, is located in the second intron immediately adjacent to that skipped exon. No coding variation in the locus is in linkage disequilibrium (LD) (*r* ^2^ > 0.20) with this variant in EUR or AFR, strongly suggesting regulatory function. The variant is thus independent of the known skin pigmentation^43^ SNP rs1426654 (a missense variant in the third exon), which is also an SRE SNP with high *F*_ST_ (=0.97) and high XP-CLR (=74). Furthermore, rs2675347 is not an eQTL based on GTEx data^34^ (v6p) in > 40 tissues, raising the possibility of splicing-specific regulatory function. No other intronic SRE SNP is in LD (*r* ^2^ > 0.20) with rs2675347 in EUR. We confirmed skipping of the second exon using GTEx data, for example in “Skin – Sun Exposed (Lower Leg) (Supplementary Figure 5)”. Furthermore, the SNP is a best sQTL (using Altrans applied to the first-phase GTEx data, −log10 (p) = 3.66 and correlation between exon-exon quantification and genotype = 0.384) although this effect on splicing requires further validation. The SNP rs28777, located in the pigmentation gene *MATP* (*SLC45A2*), has also been found to be associated with hair color^44^, and is an ISE SNP that shows extreme population differentiation between EUR and AFR (*F*_ST_ = 0.85) and is an outlier for both XP-EHH (=3.3) and XP-CLR (=136.4). Although a missense SRE (rs27622) is in modest LD (*r* ^2^ > 0.41) with rs28777 in EUR, the SNP is not population-differentiated between EUR and AFR (*F*_ST_ = 0.03). These examples suggest that alternative splicing regulation may function as a molecular mechanism of adaptation mediating the effect of selection in populations. We thus proceeded to comprehensively identify intronic SRE SNPs with high degree of population differentiation that have independent additional support from the “long-range haplotype” methods and from the test for linked selection as having been the target of recent positive selection. Notably, among the ISE SNPs with extreme population differentiation (*F*_ST_ > 0.70), we identified outlier SNPs for XP-EHH (i.e., XP-EHH > 2 or XP-EHH < −2; Supplementary Figure 6). Furthermore, confirming our claim that the population-differentiated SRE SNPs contain loci likely to be under selection, we found that the ISE SNPs showed a thicker tail-end XP-CLR distribution (Supplementary Figure 7) than non-SRE intronic SNPs across the genome.

We investigated the degree of population differentiation and the evidence for selection for the SRE SNPs that have also been identified as transcript ratio QTLs (trQTLs) (Fig. 2) in lymphoblastoid cell lines (LCLs) using RNA-Seq data from the GEUVADIS Consortium^25^. These QTLs alter the ratio of each transcript to the gene total and constitute significant genetic effects on transcript structure in this cell type. The trQTLs were identified using a univariate approach in contrast to the sQTLs associated with changes in the splicing ratios of genes and identified using the multivariate sQTLseekeR^36^ approach. The enrichment results we report above for SRE SNPs (which are defined according to disruption of hexameric motifs and thus *a priori* may preclude tissue dependence) in intronic regions hold robustly for these QTLs (which, in contrast, are likely to act in a tissue-specific manner). Indeed, relative to their non-SRE counterparts, a greater degree of enrichment (in terms of level of significance) for high *F*_ST_ (*F*_ST_ > 0.70) holds for the intronic trQTLs (P = 5.29 × 10^−14^) than for the larger set of intronic SRE SNPs (see above). Notably, among the subset of ISE SNPs that were also identified as trQTLs, we observed a slightly greater correlation (Spearman’s ρ = 0.23, P = 1.12 × 10^−12^) between *F*_ST_ and XP-CLR than found for the full set of ISE SNPs (Spearman’s ρ = 0.18). For both the ISE SNPs and the subset of trQTLs (in LCLs), this correlation is greater than for the full set of SNPs genome-wide (Spearman’s ρ = 0.12, P < 2.2 × 10^−16^). Thus, among these high-*F*_ST_ intronic trQTLs, we found robust evidence for signature of selection. For comparison with the first-phase GTEx data, the best sQTLs per exon-exon link also showed a greater correlation (Spearman’s ρ = 0.18, P < 2.2 × 10^−16^) between *F*_ST_ and XP-CLR than the full set of SNPs.

Among the trQTLs, we found 163 unique variants that have extreme *F*_ST_ (*F*_ST_ > 0.70), which represent 0.003 of all trQTLs tested; this is an order of magnitude greater than the proportion of all tested SNPs (i.e., 0.00036) with extreme *F*_ST_ (*F*_ST_ > 0.70). We observed no significant difference (Mann-Whitney U test P = 0.8039) in degree of population differentiation between the cis-eQTLs (as reported by the GEUVADIS Consortium) and the trQTLs. Among the trQTLs with high *F*_ST_ (*F*_ST_ > 0.70), we found a small number of transcripts with differential isoform usage (Mann-Whitney U test) between the European and African samples in the GEUVADIS dataset (Supplementary Table 2).

### LOF SNPs in SREs

Among LOF SNPs, both SRE and non-SRE SNPs are enriched for low *F*_ST_ (<0.05) relative to the other SNP classes (P < 2 × 10^−16^ for all comparisons), but the two types of LOF SNPs do not significantly (P = 0.85) differ from each other in enrichment for low *F*_ST_. This enrichment for low population differentiation at LOF variants in splicing motifs (relative to other SNP classes also in splicing motifs) was observed for the rarer variants (Supplementary Figure 3), and this is consistent with certain splicing regulatory motifs around LOF variants being constrained by negative selection, although we are unable to detect any difference in comparison with LOF SNPs in non-SRE regions. Of note, although no LOF SRE SNPs attain the same level of extreme population differentiation as SNPs in intronic splicing motifs, the SRE LOF variants may show *some* evidence of being more likely to attain *F*_ST_ > 0.30 (Fig. 3) than the LOF variants in the rest of genome (although the odds ratio is not significant likely due to the small number of LOF SNPs with such *F*_ST_), raising the possibility that splicing-regulatory LOF variants may, in some cases, be targeted by positive selection.

### SREs in synonymous sites are subject to purifying selection

Among rare synonymous SNPs (MAF < 0.01 in EUR), SRE SNPs have a higher odds ratio (OR = 1.023) for low *F*_ST_ compared to non-SRE SNPs (although this odds ratio is not significant) (Fig. 3). Furthermore, SRE SNPs in synonymous sites show a significantly greater level of pathogenicity (see below under “Pathogenicity of SRE variants”) than their non-SRE counterparts. Taken together, these results are consistent with greater purifying selection on synonymous SRE sites than on synonymous non-SRE sites, suggesting selective constraint on these exonic SREs to maintain their splicing function.

### Selection acting on disruption or generation of SREs

We define the *SRE-disrupting allele* as the allele that destroys the SRE motif; in the presence of this allele, the SRE variant is expected to lose its splicing function. The *SRE-inducing allele* is defined as the allele that generates the hexameric motif for the SRE. To gain further insights into the selective forces acting on splicing regulation and into the pattern of population differentiation observed at the SRE SNPs, we evaluated whether disruption or generation of the splicing motif by the derived allele is under selection in the various SNP classes.

We tested SNPs in ESE and in ESS sites (see Methods) for evidence of extreme population differentiation. We found no significant difference in degree of population differentiation between ESE and ESS variants among loss-of-function (LOF) SNPs (P = 0.20), non-synonymous SNPs (P = 0.17), and synonymous SNPs (P = 0.95). Our data indicate that the disruption of ESS and generation of ESE in synonymous sites may be influenced by positive selection. Indeed, at these loci, we observed a significantly higher proportion of population-differentiated SNPs (*F*_ST_ > 0.70) (P = 8.9 × 10^−8^ and P = 9.8 × 10^−12^ for ESS disruption and ESE generation, respectively) in comparison to the proportion expected from matched synonymous non-SRE SNPs. For example, the synonymous ESE SNP rs1189899 (with SRE-inducing allele ‘A’) is highly population-differentiated, *F*_ST = _0.8, and shows extreme XP-CLR (=52.5).

### Modeling splicing regulatory element using population differentiation, derived allele frequency and SNP functional class

We modeled the probability of SNP overlap with an SRE (see Methods) using a logistic model. In this model, the probability of SRE overlap resulted from the combination of genome-wide (e.g., population-level effects or global effects on the SNP functional class) as well as locus-specific effects. For intronic SNPs, the significant positive effect (P = 0.04) of *F*_ST_ on SRE annotation (using the derived allele frequency (DAF) as covariate) is consistent with our earlier observation of increased population differentiation for intronic SNPs in splicing motifs (relative to non-SRE SNPs). Other functional classes of SRE SNPs were not significant for higher *F*_ST_ under this model.

### Pathogenicity of SRE variants

**Figure 4:**
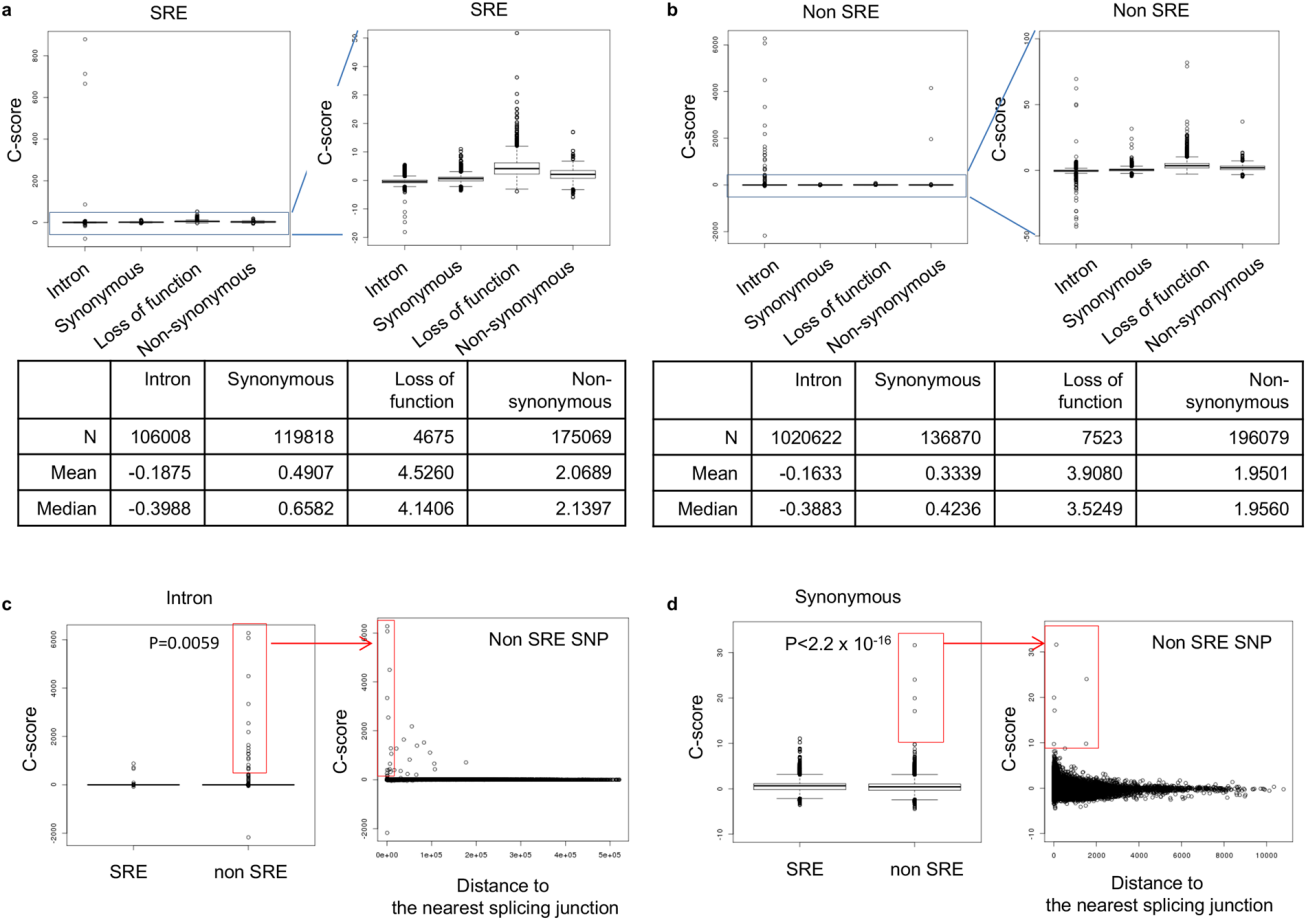
Pathogenicity of SRE SNPs in each SNP class. We used the C-score calculated from the Combined Annotation-Dependent Depletion method. (**a**) Distribution of C-score for SRE SNPs in each SNP class. (**b**) Distribution of C-score for non-SRE SNPs in each SNP class. (**c**) Thirty-four outliers with high C-score (defined as C-score > 10) at intronic non-SRE SNPs have shorter distance to the nearest splice junction than expected. A significant difference (P = 0.0059) in C-score between SRE and non-SRE SNPs within introns was observed. (**d**) Four outliers with high C-score (defined as C-score > 10) at synonymous non-SRE SNPs have shorter distance to the nearest splice junction than expected. A significant difference (P < 2.2 × 10^−16^) in C-score between synonymous SRE and synonymous non-SRE SNPs was observed. P-values shown are those obtained after excluding the outlier SNPs.

We conducted a comparison of the SRE and non-SRE SNPs using the Combined Annotation-Dependent Depletion (CADD)^45^ method. The C-score provides a metric of deleteriousness (of a SNP) that integrates diverse annotations and is correlated with pathogenicity, disease severity, and experimentally measured regulatory effects. SRE SNPs have higher C-scores than non-SRE SNPs among coding variants (Fig. 4a and b). Intronic SNPs in general tend to have lower C-scores than other SNP classes. On closer inspection, we found outliers with high C-score (defined as C-score > 10) at non-SRE SNPs. Particularly, 35 and 5 of these were intronic and synonymous SNPs respectively and, notably, were much closer to the nearest splice junction than expected (Fig. 4c and d).

### SRE SNPs are found among top disease associations identified by genome-wide association studies

**Figure 5:**
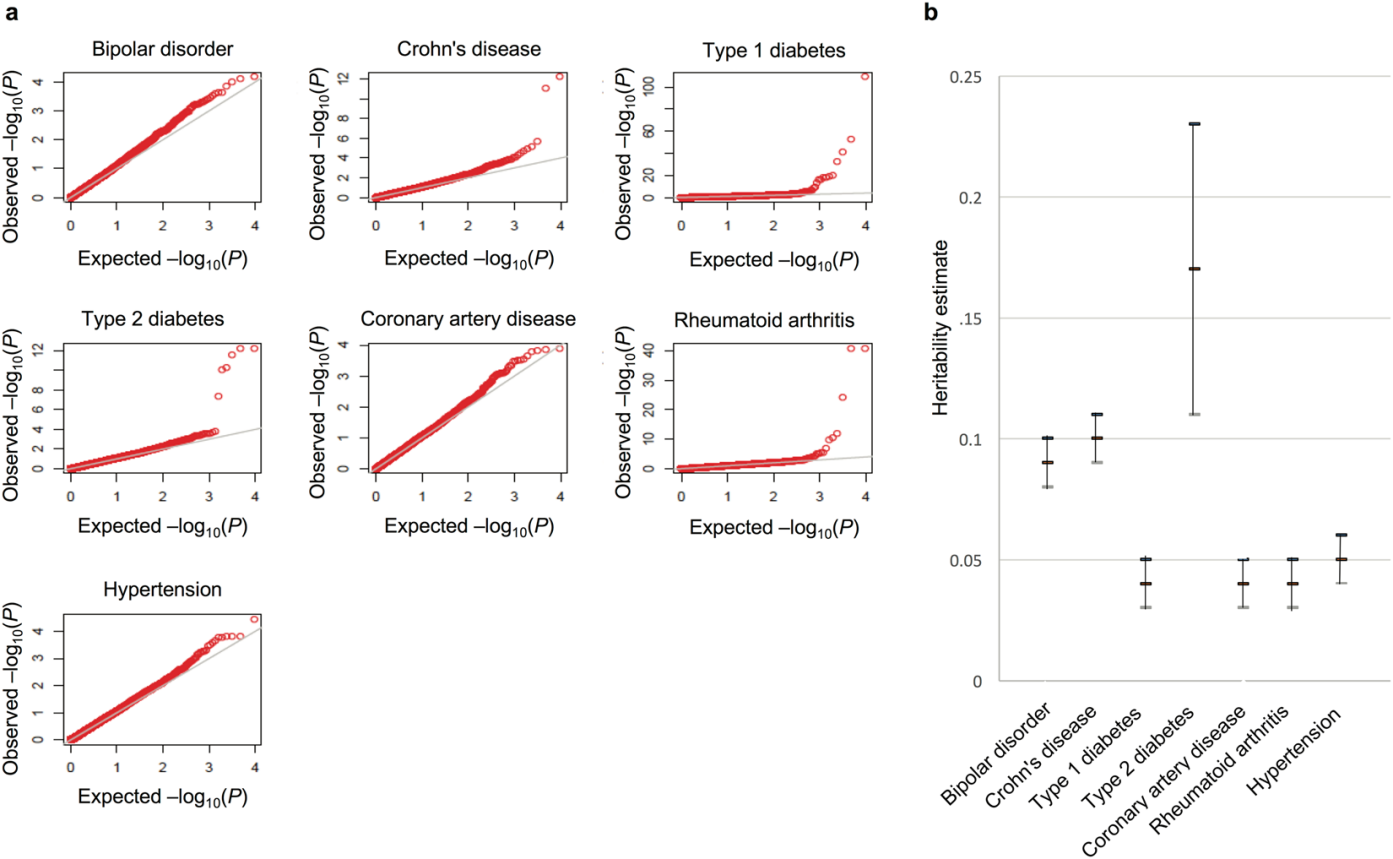
SRE SNPs and mapping disease-associated loci. (**a**) SRE SNPs were found among the top disease associations with the WTCCC disease traits. Several genome-wide significant (Bonferroni-adjusted p < 0.05) associations with disease traits were found to be SRE SNPs. The Q-Q plots show the distribution of SRE p-values for association with the disease phenotypes. (**b**) The contribution of the SRE SNPs to trait heritability varies with the trait, which may indicate the relative importance of splicing regulation to the genetic architecture. The estimate for the heritability and the corresponding standard error are shown.

To determine whether the SRE annotation is useful for identifying disease-associated variants, we evaluated the GWAS of 7 complex disorders from the Wellcome Trust Case Control Consortium (WTCCC). We found SRE SNPs among the very top associations with some WTCCC disease phenotypes, including those meeting genome-wide significance (Bonferroni-adjusted P < 0.05) (Fig. 5a). Indeed, SRE SNPs were among the most significant associations not only in the autoimmune disorders (such as previously shown using eQTL mapping in lymphoblastoid cells and whole blood)^46, 47^ but also, intriguingly (because our definition of SRE is, initially, non-tissue-dependent), in type 2 diabetes (a disease which implicates multiple tissues, including beta cells and insulin-responsive peripheral tissues such as adipose, muscle and liver). For the autoimmune disorders and type 2 diabetes, SRE SNPs were substantially more enriched for low p-values than non-SRE SNPs (Kolmogorov-Smirnov test p < 0.05). The enrichment for low p-values for the autoimmune disorders and type 2 diabetes was confirmed by using random sets of non-SRE SNPs matched on MAF, extent of LD, gene size, and distance to exon/intron boundary versus a uniform distribution as the null. All novel association signals identified among the SRE SNPs were intronic.

To estimate the contribution of the SRE SNPs to trait variation, we implemented a mixed-effects model with the total genetic effect from either the SRE or non-SRE SNPs modeled as a separate random effect (see Methods). Among the immune-related and inflammatory disease traits, we found disease-dependent SRE-based heritability estimates: Crohn’s disease (0.10 ± 0.01), type 1 diabetes (0.04 ± 0.01), and rheumatoid arthritis (0.04 ± 0.01). Interestingly, bipolar disorder (0.09 ± 0.01), coronary artery disease (0.04 ± 0.01), hypertension (0.05 ± 0.01), and type 2 diabetes (0.17 ± 0.06) also showed significant contribution to trait heritability, at varying levels, from the SRE SNPs (Fig. 5b). We note these GWAS studies have similar sample sizes and the same SRE SNPs were being evaluated. Thus, the differences in the heritability estimates from the SRE SNPs among these phenotypes are notable and may suggest the relative importance of splicing regulation to the genetic architecture of these disease traits.

We highlight some examples of population-differentiated GWAS loci, the impact of alternative splicing on the resulting protein domain, and the evidence for selection.

### Case studies of population-differentiated SRE SNPs

#### Pharmacogenetic Locus: rs2239121 or rs216013 in CACNA1C

**Figure 6:**
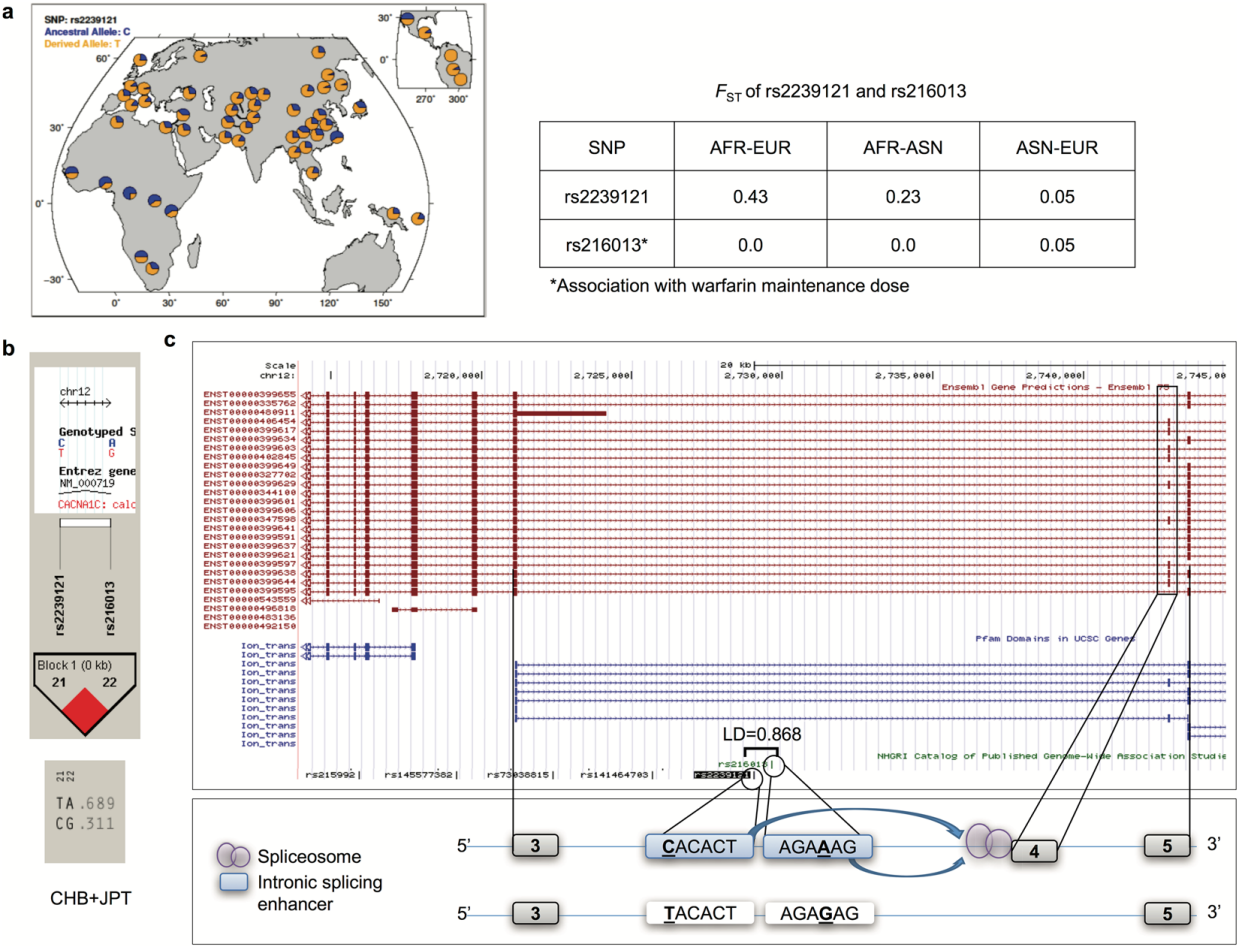
Pharmacogenetic variant rs2239121 in *CACNA1C*. (**a**) Geographical distribution of allele frequency of the SRE-disrupting allele T. rs2239121 is highly differentiated between the AFR and EUR population, but rs216013 is not differentiated among populations. The map was generated using the HDGP Selection Browser: http://hgdp.uchicago.edu/tmp1/Alfreqs/rs2239121.frqs.pdf ^85^. (**b**) Linkage disequilibrium and haplotype analysis for rs2239121 and rs216013. (**c**) Model of exon skipping affected by rs2239121, rs216013 in intronic splicing enhancer and impact of the skipped exon on protein domain region.

The variants rs2239121 and rs216013, located in the pharmacogenetic (“VIP”) gene *CACNA1C*, have the C/T and A/G alleles, respectively. The derived T allele at rs2239121 and the A allele at rs216013 are the SRE-disrupting alleles. The SNPs are in strong LD (r^2^ = 0.868 in EUR) and rs216013 has been found to be associated with warfarin maintenance dose in patients of European descent^48^. This clinical trait shows substantial population divergence between populations^49, 50^. Rs2239121 is population-differentiated, but the reported variant rs216013 is not (Fig. 6a). The normalized XP-EHH score for rs2239121 is 2.3 (i.e., at the 98.9 percentile genome-wide). The frequency of the T (i.e., SRE-disrupting) allele at rs2239121 varies with the continental population (with the frequency lowest in AFR and highest in the ASN) (Fig. 6c). The difference in derived allele frequency between EUR and AFR is ~0.50. Interestingly, the rs2239121 T allele and the rs216013 A allele are frequently (0.689) found on a single haplotype in the Asian HapMap populations (CHB/JPT) (HapMap version 2, HaploView^51^) (Fig. 6b). Moreover, there is putative exon skipping adjacent to these SRE SNPs, and this skipped exon is a part of the region encoding a voltage-dependent L-type calcium channel subunit alpha-1C domain (VDCCAlpha1) in *CACNA1C* (Fig. 6c). Interestingly, the gene is also reported to play a role in the pathogenesis of psychiatric disorders, including bipolar disorder and schizophrenia^52, 53^.

#### Disease Susceptibility Locus: rs4506565 in TCF7L2

**Figure 7:**
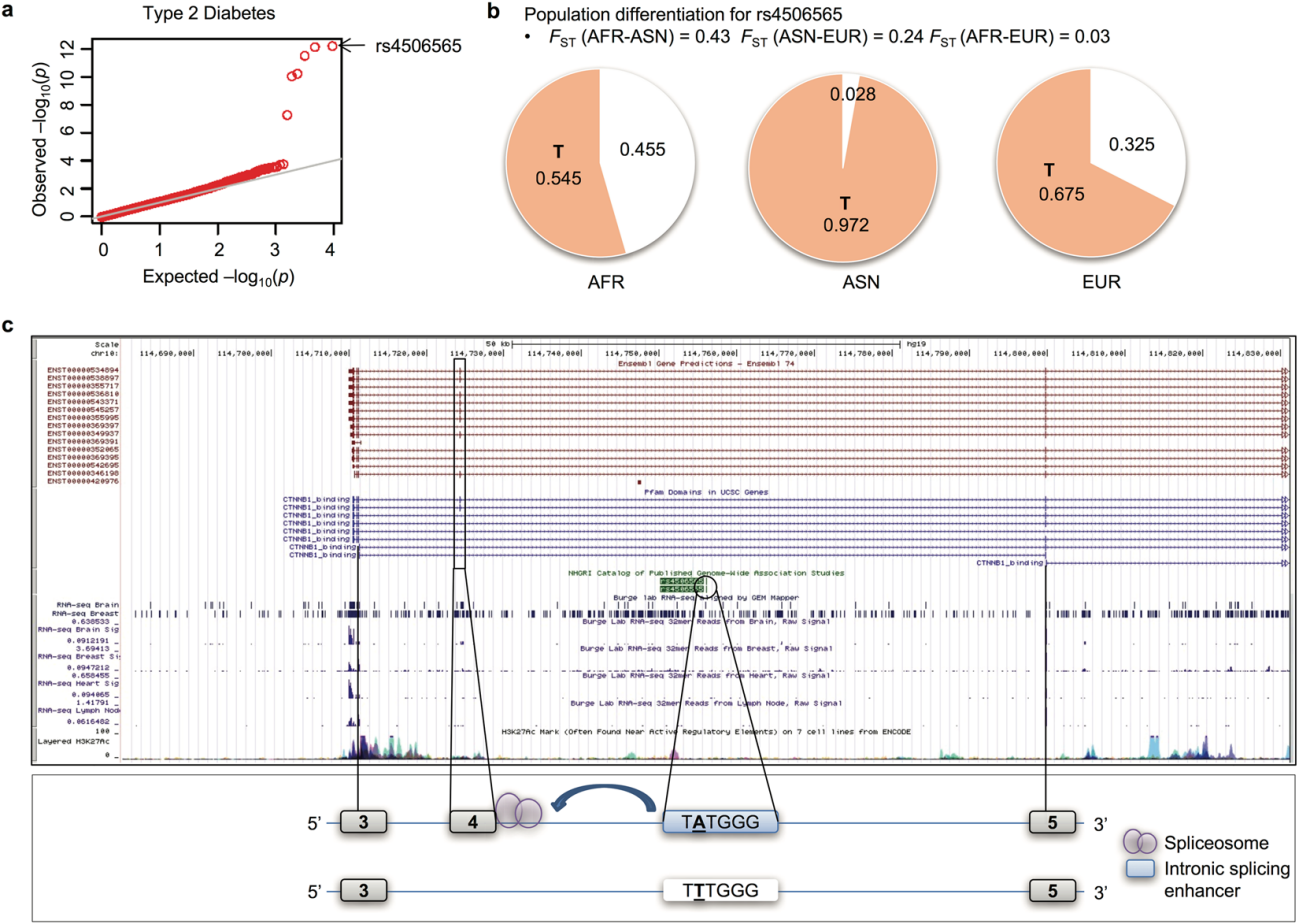
Disease susceptibility variant rs4506565 in *TCF7L2*. (**a**) Significant association with Type 2 Diabetes. (**b**) rs4506565 is highly differentiated among populations and shows the highest *F*_ST_ = 0.43 between AFR and ASN populations. (**c**) Model of exon skipping affected by rs4506565 in intronic splicing enhancer and impact of the skipped exon on protein domain region.

The SNP rs4506565 is a T/A variant with T the ancestral allele. As shown in Fig. 7, the T allele frequency shows population variation, tracking the continental groups with frequency highest in ASN, intermediate in EUR, and lowest in AFR. The difference in derived allele frequency between ASN and AFR is 0.43. The normalized XP-EHH score (between CHB and YRI) is 2.20 (i.e., at the 98.6 percentile genome-wide). The T allele is predicted to be the SRE-disrupting allele and is known to confer risk to type 2 diabetes^54, 55^. Furthermore, there is strong evidence of exon skipping adjacent to this SRE SNP with important consequence for protein function. The putatively skipped exon is part of the region translated into a CTNNB1-binding domain^56, 57^ (Fig. 7c). *TCF7L2* is a transcription factor and a cancer-related gene^58–60^.

No coding variation is in LD (at r^2^ > 0.20) with rs4506565 in EUR, AFR or ASN, strongly suggesting regulatory function for this GWAS locus. We considered SNPs in strong LD (r^2^ > 0.80 in EUR) with the index SNP rs4506565. We identified 8 SNPs, all of which were intronic; of these, only one SNP (rs7901695) (a) showed a similar level of population differentiation and of significant association with type 2 diabetes (using a large-scale meta-analysis^61^) as the index SNP rs4506565 and (b) was also an SRE SNP that was immediately adjacent to the skipped exon. Rs4506565 was the more significant association. Neither rs7901695 or rs4506565 was detected as an eQTL by GTEx data^34^ in >40 tissues, raising the possibility of splicing-specific regulatory function. Interestingly, rs4506565 (but not rs7901695) shows a nominally significant effect on transcript relative expression of *TCF7L2* (p = 0.04) from sQTLseekeR first-phase GTEx data, although clearly this requires additional functional validation.

### Selection at SRE sites conditional on background selection and other features: sensitivity analysis

We evaluated the effect of “background selection”^62–64^ (Supplementary Information) on our enrichment analyses for extreme population differentiation. Background selection induces the purging of non-deleterious alleles by virtue of physical proximity to deleterious alleles. Variants from the various SNP classes that overlap SREs show different patterns of background selection (Supplementary Figure 8). Low values of background selection indicate near complete reduction in nucleotide diversity and high values indicate a small reduction. In our enrichment analyses, we sought to account for any reduction in nucleotide diversity at a *neutral* site attributable to purifying selection at a nearby locus. We defined a “Cochran-Mantel-Haenzel” procedure (see Supplementary Methods) from “strata” of background selection on the genome, setting up a series of 2 × 2 contingency tests (on the independence of high *F*_ST_ and the SRE designation) and computing a “common odds ratio” (from the individual odds ratios across the strata) that provides a quantification of the association that has been adjusted for the potential confounding effect of background selection. In introns, SRE SNPs show a significantly greater amount of reduction in diversity than non-SRE SNPs (P < 2.2 × 10^−16^). We found that the enrichment of high-*F*_ST_ SNPs observed, in intronic regions, among SRE SNPs relative to non-SRE SNPs held robustly (P = 1.8 × 10^−4^, common OR = 1.2) after accounting for background selection (see Methods). Indeed, the use of the Cochran-Mantel-Haenzel procedure appears to result in improved power for detecting the greater effect of selection on SRE SNPs relative to non-SRE SNPs. We also found that without explicitly accounting for background selection, we may get an inflated estimate of odds ratio (e.g., for 0.8 < *F*_ST_ ≤ 0.9, OR = 1.42, in introns) that does not reflect the odds ratio in the individual strata.

We also tested the sensitivity of the results of the comparisons between SRE SNP and non-SRE SNPs within each functional class to potential confounding due to MAF, gene size, distance from exon/intron boundary, and extent of LD. We find no significant difference (Mann-Whitney U test P > 0.05 for all comparisons) for each such feature between SRE and non-SRE SNPs. Using a random sampling approach that uses randomly selected non-SRE SNPs (n = 1000 sets) matched on these features, we confirmed that the intronic SRE SNPs were significantly enriched for high-*F*_ST_ SNPs (permutation p-value < 0.001).

## Discussion

We performed a whole-genome scan of genetic variants in splicing regulatory elements with high differentiation between populations. We hypothesized that variants associated with alternative splicing in regulatory elements may present candidate loci for signatures of selection. We investigated the pathogenicity of SRE variants as well as tested for signatures of selection at these loci using multiple approaches, such as those that consider haplotype diversity and structure as well as multi-locus differentiation.

We found that SRE SNPs are enriched for disease-associated variants. Different SNP classes, defined with respect to physical location or type of amino acid change, among the SRE variants were found to have different patterns of population differentiation relative to their non-SRE counterparts. An analysis of the overlap of intronic SRE SNPs (which show significant enrichment for high population differentiation relative to the remaining [non-SRE] intronic SNPs) with those identified by high-throughput sequencing of the transcriptome^25^ as transcript ratio QTLs demonstrated an even more significant enrichment, suggesting that the use of context annotation (e.g., tissue) for regulatory variation may well improve detection of signature of selection. Although LOF variants in splicing motifs are enriched for low population differentiation and low frequency alleles relative to other SNP classes, consistent with the effect of purifying selection, we also observed some evidence of enrichment for population-differentiated SNPs relative to LOF non-SRE SNPs (although a more robust test is required due to the small number of population-differentiated LOF variants overall), suggesting that some of these splicing-associated variants may well have contributed to local adaptation in human populations.

The observed excess of extreme population differentiation at intronic SRE SNPs is consistent with recent positive selection on some of these loci, on the basis of haplotype-based tests (iHS and XP-EHH). A well-known example of such a gene under positive selection (namely, *SLC24A5*, a member of the potassium-dependent sodium/calcium exchanger family that has been shown to be involved in skin pigmentation) was, in our data, implicated by a positively selected SRE variant. We implemented a Cochran-Mantel-Haenzel estimator of the effect of positive selection to account for “background selection”^62, 65^ and observed a highly robust enrichment for population-differentiated SNPs among the intronic SRE SNPs. This approach also allowed us to quantify potential inflation in the effect of selection on these variants that is actually due to background selection. In short, we found support for splicing regulation as a molecular mechanism that may mediate the effect of selection; the SRE variants should therefore provide, for future studies, candidate loci potentially targeted by selection.

To gain further insight into the functional role of the SRE SNPs, we performed a comparison of the pathogenicity of these variants relative to their non-SRE counterparts through a recently proposed metric^45^ that combines multiple annotations. We found a significantly greater C-score for SRE than non-SRE variants among coding SNPs (P = 1.57 × 10^−9^), suggesting the utility of the SRE annotation for detecting pathogenicity. Interestingly, the non-SRE variants with the highest C-score were found to be significantly closer to the nearest the splice junction than expected.

Regulatory variation is likely to be an important source of human phenotypic diversity, including variation in disease risk. Yet, relatively little is known about the adaptive significance of regulatory variation and even less is known about the relative contribution of regulatory and coding variation. The approach we used here – testing splicing-associated variation within each coding SNP class – enabled us to explore to what extent SREs are potential targets of positive selection and to what degree purifying selection has constrained patterns of variation at these regulatory loci, independently of any selective effects on the particular coding SNP class. We showed that splicing regulatory elements are important contributors to differentiation between populations and that regulation of transcript diversity through splicing in some key genes may be under selection.

Recent studies have contributed to the characterization of transcriptome variation, mostly understood as the measurement of the diversity of transcripts and differences in gene expression across tissues and cell types, or between diseased tissues and healthy ones^34, 66^. Furthermore, some studies have investigated expression variability between human populations^67, 68^. Genotype-dependent expression of a specific exon or transcript isoform ratio is important information for understanding the phenotypic effects of splicing^36, 69^. To extend studies of transcriptome variability in human populations, our study evaluated the extent to which regulatory SNPs affecting splicing show evidence for selection, demonstrating that SRE SNPs may be an important contributor to human population divergence. These sequence variations in SREs exert their regulatory (and downstream phenotypic effects) by potentially disrupting or activating the function of the regulatory motifs. Skipped exons from this regulatory process may lead to different versions of a protein suited to specific environments^70^. Although our analyses are restricted to the most prevalent form of AS event – exon skipping – and single substitutions in SREs (versus more complex disruptions), a primary contribution of this work is to present a methodology that uncovers evidence for selection at loci enriched for regulatory function in the genome and demonstrates their relevance for genome-wide association studies of diseases and pharmacologic phenotypes. This study also contributes to the understanding of the complex molecular processes underlying phenotypic differences in human populations. Disruptions in SREs may cause errors in RNA splicing or its regulation, providing functional characterization for loci that have been reported for a variety of heritable human diseases^71^. Furthermore, our study has implications for pharmacogenomics in diverse human populations (i.e., pharmacoethnicity) and for precision medicine^49, 50^, enabling studies of differences, for example, in drug metabolism^72–74^ or resistance to chemotherapeutic agents^75–77^. We anticipate that the annotation and methodology provided here will be useful for characterizing the genetic basis of disease risk and therapeutic response.

## Methods

### Identifying SNPs within SRE sites associated with exon skipping events

We have previously published methods for identifying intronic SNPS within SRE sites associated with exon skipping events^78^. For this study, we used 2,130,021 intronic SNPS within SRE sites from an updated version of dbSNP137 and the human genome build GRCh37. Coding SNPs were classified functionally following TGP’s annotation (here, called “SNP classes”): “synonymous”, “non-synonymous”, and “loss-of-function”. To identify coding SNPs in ESE or ESS sites, we carried out the same procedures previously described, in our recent study, for ISE SNP identification^78^, this time using 979 ESE^4^ and 496 ESS hexamers^79^ derived from a neighborhood inference algorithm, which were obtained from Table S1 (“NI Scores for All Hexanucleotides”) of http://dx.doi.org/10.1371/journal.pgen.0020191)^80^. Briefly, the sequence context around a coding variant (5 bases upstream and downstream) was extracted using the twoBitToFa command (https://genome.ucsc.edu/goldenPath/help/twoBit.html). From this 11-base sequence, we identified all possible 6-mer motifs that include the coding variant by taking a 6-base long window with the SNP in the last position and successively shifting until the SNP is in the first position. A coding SNP was considered an ESE or ESS SNP when the sequence of the exonic hexamers surrounding the coding SNP exactly matched one of the ESE/ESS motifs. For the predicted ESE/ESS SNPs, using the genomic coordinates of AS transcript isoforms, we confirmed that the exon embedding the given coding SNP is skipped; this analysis identified 177,556 ESE/ESS SNPs.

We also overlapped the SRE SNPs with the transcript ratio QTLs (trQTLs) identified in LCL RNA-Seq data from the GEUVADIS project^25^. In analyses of population differentiation, we used these trQTLs detected in the CEU population at FDR < 0.10. For additional support for the effect of the SRE SNPs on splicing, we tested for enrichment of SRE SNPs among the best sQTLs for exon-exon link from the first-phase GTEx data^34^ in 9 human tissues while matching on MAF, gene size, distance to exon/intron boundary, and extent of LD (n = 1000 random sets). We also considered the distribution of p-values from the associations with splicing ratios of genes in GTEx whole blood to test for enrichment for low p-values among the SRE SNPs relative to randomly generated sets (n = 1000) of SNPs matched on the SNP attributes. Throughout, when matching on MAF and extent of LD for enrichment analyses or for the comparisons, we used the data in EUR.

### Testing SRE SNPs in genome-wide association studies

We tested whether our SRE annotation would enable the identification of disease associations with improved false discovery rate. Towards this end, we generated a Q-Q plot for each WTCCC disease (bipolar disorder [BD], coronary artery disease [CAD], hypertension [HT], type 1 diabetes [T1D], type 2 diabetes [T2D], crohn’s disease [CD], rheumatoid arthritis [RA]) using the association p-values of the SRE SNPs. A leftward shift from the diagonal line would indicate a departure of the observed distribution from the uniform distribution. We compared the distribution of p-values for the SRE SNPs and the non-SRE SNPs using the Kolmogorov-Smirnov test. We also identified the SREs among the genome-wide significant disease associations (Bonferroni-adjusted p < 0.05). We evaluated the extent to which MAF, extent of LD, gene size, and distance to exon/intron boundary may be confounding enrichment results by using as control 1000 randomly generated sets of non-SRE SNPs matched on these attributes.

We implemented a mixed-effects model to estimate the proportion of disease risk variance explained by the SRE SNPs (Eq. 1–2):

1 $$Y=Xb+{G}_{SRE}+{G}_{non-SRE}+e$$

2 $$var(Y)={A}_{SRE}{\sigma }_{SRE}^{2}+{A}_{non-SRE}{\sigma }_{non-SRE}^{2}+I{\sigma }_{e}^{2}$$

Here *b* is a vector of fixed effects; *A*_*SRE*_ and *A*_*non–SRE*_ are the genetic relatedness matrices calculated from the SRE and non-SRE SNPs, respectively; and *G*_*SRE*_ and *G*_*non–SRE*_ are the random genetic effects attributable to the SRE and non-SRE SNPs, respectively; and *e* is the residual. The variances ${\sigma }_{SRE}^{2}$ and ${\sigma }_{non-SRE}^{2}$ were estimated using restricted maximum likelihood^81^, allowing us to estimate the contribution to heritability of the SRE SNPs as the proportion of trait variance explained by this special class of SNPs^82^, $\widehat{{\sigma }_{SRE}^{2}}/\widehat{{\sigma }_{Y}^{2}}$.

### Estimating population differentiation

*F*_ST_, the fixation index, is a measure of population differentiation. For *F*_ST_ estimation between populations, we downloaded genotype data for the 4 “super” populations from the 1000 Genomes Project: 1) AFR (the merge of the African subpopulations of ASW, YRI, and LWK), 2) EUR (the merge of the European subpopulations of IBS, CEU, GBR, FIN, and TSI), 3) ASN (the merge of the East Asian subpopulations of CHS, JPT, and CHB). *F*_ST_ was calculated for each SNP using the allele frequencies estimated from the unrelated individuals for the populations under comparison. We used the Weir and Cockerham (unbiased) estimator for *F*_ST_^83^ (See Supplementary Information).

### Assigning allele with derived or ancestral status

We compiled the SNPAncestralAllele.bcp and Allele.bcp data downloaded from the dbSNP FTP site. The ancestral allele and derived allele annotations were derived from comparison of human DNA to chimpanzee DNA based on a previously published method^84^.

### Calculating SNP allele frequencies

We used the allele frequency information from each of the four populations: AFR, AMR, ASN, ad EUR from TGP.

### Annotating genetic variation with C-score

We utilized the publicly available data on C-score (v1.0) of human genetic variation, which is an integrative measure of functionality and pathogenicity, to annotate SRE and non-SRE SNPs. We did a non-parametric (Wilcoxon) comparison of the SRE and non-SRE SNPs in each of the SNP classes.

### Assessing statistical significance

For each functional SNP class, we used the Mann-Whitney U test to compare the SRE SNPs and non-SRE SNPs for enrichment for low *F*_ST_ as well as for high *F*_ST_, the derived allele frequency between the SRE SNPs and genomic background (defined using all derived alleles in dbSNP) and the C-score between the SRE SNPs and non-SRE SNPs. We also investigated the degree of population differentiation among the trQTLs identified in LCL RNA-Seq data^25^.

For each SNP class, we calculated the odds ratio *OR*(*F*;*S*) as follows (Eq. 3):

3 $$OR(F;S)=\frac{P(F|S)}{1-P(F|S)}/\frac{P(F{|S}^{c})}{1-P(F{|S}^{c})}$$

Here *F* is an *F*_ST_ bin (such as in an *F*_ST_ bin-matched comparison of SRE and non-SRE SNPs) or a flag for extreme *F*_ST_ (either *F*_ST_ > 0.70 or *F*_ST_ < 0.05), *S* is a SNP set (SRE SNPs in a given SNP class) and *S* ^*c*^ is the complement set in the SNP class. *P*(*F|S*) is the probability of *F* given *S*.

We also considered the odds ratio *OR*(*F*; *S*, Δ), which conditions on a set of features, Δ, such as the derived allele frequency *D* (calculated using the EUR samples) or the background selection B-value; this is defined as in *OR*(*F*; *S*) with the conditional probability *P*(*F|S*) replaced by *P*(*F|S*, Δ). For example, in the case of low *F*_ST_ SNPs in a fixed SNP class, this odds ratio, with Δ consisting of *D*, would test whether the larger proportion of SNPs with low population differentiation among SRE SNPs relative to non-SRE SNPs holds for those SNPs with low DAF.

A p-value was generated for these comparisons using the derived 2 × 2 contingency table.

For the (non-parametric) comparisons between SRE and non-SRE SNPs, wilcox.test as implemented in R (http://http://www.r-project.org/) was used.

### Modeling the SRE overlap

We modeled the probability of SRE overlap in the various SNP functional classes. For a (genic) SNP *i* in a given SNP class, we modeled *p*_*i*_, the probability of SRE overlap, as follows (Eq. 4):

4 $${{\rm{p}}}_{{\rm{i}}}=1/(1+\exp (-({{\rm{\beta }}}_{0}+{\rm{\beta }}\cdot {\rm{\Delta }})))$$

where β is a vector of effect sizes and Δ is a set of features such as the derived allele frequency *D*, the *F*_ST_ *F*, the background selection value (B-value) *B*, and extent of LD with the SNP’s neighbors, ${\rm{L}}={\sum }^{}{{\rm{r}}}_{{\rm{j}}}^{2}$. Here β · Δ denotes the weighted sum of the features. β_0_ can be seen as a genome-wide (global) effect (such as due, in part, to demographic processes) whereas β captures locus-specific effects on the SRE annotation.

### Empirical P-value for enrichment

We also empirically tested for enrichment of high-*F*_ST_ SNPs among the SRE SNPs after conditioning on the DAF, the B-value, and the extent of LD as well as the number of SRE SNPs tested. B-values and DAF (calculated from EUR) were binned (of width 100 and 0.05, respectively) while extent of LD (also generated from EUR) was binned into the intervals [0, 50), [50, 85), [85, 110), [110, 140), and ≥140. For an empirical null distribution, 1000 sets of randomly chosen SNPs were generated that match the B-value, LD extent, and DAF of the SRE SNPs. P-value was calculated as the proportion of null sets that matched or exceeded the observed number of high-*F*_ST_ SNPs among the SRE SNPs.

### iHS, XP-EHH, XP-CLR, and SDS

We annotated the population-differentiated SNPs (*F*_ST_ > 0.70) with the results from tests of selection using haplotype-based methods, namely, the integrated Haplotype Score (iHS)^31^ and the Cross Population Extended Haplotype Homozygosity (XP-EHH)^40^, and using an approach that considers the multilocus frequency differentiation between populations, namely, the Cross Population Composite Likelihood Ratio (XP-CLR) test^41^.

The iHS is defined as the log-transformed ratio of the integrated extended haplotype homozygosity (EHH) score for the ancestral allele-containing haplotypes to that for the derived allele containing-haplotypes in a given population (Eq. 5):

5 $$iHS=\,\mathrm{log}(\frac{\int EH{H}_{ancestral-allele}(\eta )d\eta }{\int EH{H}_{derived-allele}(\eta )d\eta })$$

The iHS is standardized to the standard normal distribution *N*(0, 1). Those SRE SNPs with |*iHS*| ≥ 2 were highlighted. XP-EHH assumes two populations (P1 and P2) and is defined, for a given allele, as the log-transformed ratio of the EHH score in population 1 to that in population 2 (Eq. 6):

6 $$XP-EHH=\,\mathrm{log}(\frac{\int EH{H}_{P1}(\eta )d\eta }{\int EH{H}_{P2}(\eta )d\eta })$$

Again, XP-EHH is standardized to *N*(0, 1). The comparison between the ancestral allele- and derived allele- containing haplotypes in the same population as defined in iHS ensures the same genomic context whereas the comparison between two populations in XP-EHH controls for local variation in recombination rates.

For XP-CLR, the allele frequencies at a neutral SNP in the two populations P1 and P2 are modeled by a (time-reversible) Wiener process from a shared ancestral allele frequency *p*_0_ (Eq. 7):

7 $${p}_{i} \sim N({p}_{0},\omega (1-{p}_{0}){p}_{0})$$

Here $\omega ={t}_{P1P2}/2{N}_{e}$ is meant to capture the population histories from the ancestral population to the present; the two populations are assumed to split from each other *t*_*P*1*P*2_ generations ago and *N*_*e*_ is the effective size of population *i*. For SNPs linked to a beneficial allele that has undergone selective sweep in one population, a composite likelihood ratio (CLR) is defined at *k* contiguous markers (Eq. 8):

8 $$CLR=\prod _{1}^{k}{L}_{k}$$

where *L*_*k*_ is the marginal likelihood at the k-th SNP. A likelihood ratio statistic is then defined.

We used a simple approach whereby the *F*_ST_ was used to prioritize SNPs, with subsequent selection scan from one or more of the haplotype-based and multi-locus tests to identify robust signatures of selection. One can apply principal component regression given the correlation of the tests. Another approach is to consider the composite likelihood at a variant *x* using the probability density of the cross-population metrics (Eq. 9):

9 $$L(x)={f}_{F{\rm{ST}}}{({x}_{F{\rm{ST}}})}^{\alpha }{f}_{XP-EHH}{({x}_{XP-EHH})}^{\beta }{f}_{XP-CLR}{({x}_{XP-CLR})}^{\gamma }$$

The exponents in the composite likelihood are suitable weights to account for the correlations of the signatures.

The Singleton Density Score (SDS) detects very recent selection, which alters the ancestral genealogy of sampled haplotypes and leads to shorter terminal branches for a favored allele. The approach provides a way to detect polygenic selection that results in subtle allele frequency shifts at a large number of loci. We compared the distribution of SDS scores for the SRE SNPs with that for the full set of SNPs using the Mann-Whitney U test to test for polygenic shifts.

## Electronic supplementary material

Supplementary Information
