# Supplementary material for: Evidence of selection on splicing-associated loci in human populations and relevance to disease loci mapping
Source: Sci Rep. 2017 Jul 20;7:5980. doi: 10.1038/s41598-017-05744-9 (PMC5519721; doi:10.1038/s41598-017-05744-9)
Supplement: Supplementary file 1 — Supplementary Information [file 41598_2017_5744_MOESM1_ESM.pdf]

Supplementary Information

Evidence of selection on splicing-associated loci in human populations and relevance to disease loci mapping

Eric R. Gamazon[1,2,4*], Anuar Konkashbaev[1,4], Eske M. Derks[5], Nancy J. Cox[1,4], Younghee Lee[3,4*]

[1] Division of Genetic Medicine, Department of Medicine, Vanderbilt University, Nashville, TN, 37235 USA

[2] Academic Medical Center, Department of Psychiatry and Department of Clinical Epidemiology, Biostatistics and Bioinformatics, University of Amsterdam, Amsterdam, The Netherlands

[3] Department of Biomedical Informatics, School of Medicine, University of Utah, Salt Lake City, UT, 84108 USA

[4] Section of Genetic Medicine, Department of Medicine, The University of Chicago, Chicago, IL, 60637 USA

[5] Translational Neurogenomics Group, QIMR Berghofer, Brisbane, QLD, 4006 Australia

Supplementary Methods

- S1 1. Estimating population differentiation
- S1 2. Background selection

Supplementary Figure

- Supplementary figure 1. Global F_{ST} distribution of SRE SNPs in each SNP class for AFR-EUR comparison
- Supplementary figure 2. Global F_{ST} distribution of SRE SNPs in each SNP class for the ASN-EUR and AFR-ASN comparisons
- Supplementary figure 3. Proportion of derived alleles among the SRE SNPs with low F_{ST} ($F_{ST} < 0.05$)
- Supplementary figure 4. Relative proportion of SRE SNPs as a function of level of population differentiation (F_{ST})
- Supplementary figure 5. GTEx transcript view showing exon skipping of SLC24A5 in Skin – Sun Exposed (Lower Leg) tissues
- Supplementary figure 6. Correlation between F_{ST} and XP-EHH
- Supplementary figure 7. Distribution of XP-CLR for intronic SRE (ISE) SNPs and intronic non-SRE SNPs
- Supplementary figure 8. Patterns of background selection for SRE SNPs in each SNP class

Supplementary Table

- Supplementary table 1. The number of SRE and non-SRE SNPs by functional category
- Supplementary table 2. Among the trQTLs with high F_{ST} ($F_{ST} > 0.70$), listed here are the transcripts with differential isoform usage (Mann-Whitney U test) between the European and African samples in the GEUVADIS dataset

SI 1. Estimating population differentiation

F_{ST}, the fixation index, is a measure of population differentiation. For F_{ST} estimation between populations, we downloaded genotype data for the 4 "super" populations from the 1000 Genomes Project: 1) AFR (the merge of the African subpopulations of ASW, YRI, and LWK), 2) EUR (the merge of the European subpopulations of IBS, CEU, GBR, FIN, and TSI), 3) ASN (the merge of the East Asian subpopulations of CHS, JPT, and CHB). F_{ST} was calculated for each SNP using the allele frequencies estimated from the unrelated individuals for the populations under comparison. We used the Weir and Cockerham (unbiased) estimator for F_{ST} [1].

$$F_{ST} = {(MSP - MSG)}\Big/{(MSP + (n_c - 1)MSG)}$$

where

$$n_c = \left(\frac{1}{r-1}\right)\left(\sum n_i - \frac{\sum {n_i}^2}{\sum n_i}\right)$$

$$MSP = \left(\frac{1}{r-1}\right) * \sum(n_i(p_i - p)^2)$$

$$MSG = \left({1}\Big/{\sum(n_i - 1)}\right) \sum n_i p_i (1 - p_i)$$

Here, *MSP* and *MSG* denote two mean square errors (for between populations and for loci within populations, respectively) [2], n_i is sample size in population i, r is the number of populations, p_i is frequency of the given allele in population i, and p is the average frequency of the allele across all populations.

SI 2. Background selection

We tested for enrichment of population-differentiated SNPs among the SRE SNPs. However, such enrichment analysis is subject to the potentially confounding effect of background selection, which reduces nucleotide diversity at a neutral site as a result of purifying selection at an adjacent site. We

therefore evaluated the robustness of the results from the enrichment analyses by taking into account the effect of background selection [3]. We assigned background selection coefficients to the TGP SNPs by mapping the genomic positions to the genomic segments and the corresponding background selection coefficients on these segments, as generated by McVicker *et al.* [3], Hg18 coordinates of the genomic segments in the original data were mapped to their corresponding Hg19 coordinates.

For analyses involving population-differentiated SNPs (e.g., $F_{ST} > 0.70$), we also considered the odds ratio $OR(F; S, D, B)$, defined as in the section above, using the probabilities $P(F|S, D, B)$ and $P(F|S^c, D, B)$, where B is the measure of background selection.

Background selection coefficients or B-values (defined on a scale of 1-1000) were binned, with each bin of width 100; TGP SNPs were assigned to these bins.

Furthermore, for a given SNP class, we calculated the following "Cochran-Mantel-Haenzel" estimator, which adjusts for the effect of background selection:

$$OR(F; S, D, B) = \frac{\sum_k N(F|S, D, B_k)N(F^c|S^c, D, B_k) / N_k}{\sum_k N(F^c|S, D, B_k)N(F|S^c, D, B_k) / N_k}$$

Here *N()* is the count function, F and F^c are the values of a binary indicator of high population differentiation, and N_k is the count in the B_k-stratum (i.e., the k-th background selection-defined bin):

$$N_k = N(F|S, D, B_k) + N(F^c|S^c, D, B_k) + N(F^c|S, D, B_k) + N(F|S^c, D, B_k)$$

$OR(F; S, D, B)$ is, in effect, a "common odds ratio" defined from the estimates of odds ratios in the various strata. The global null hypothesis, in the Cochran-Mantel-Haenzel test, is equivalent to *each* odds ratio (from a stratum) being equal to 1:

H_0: $OR_{B_k} = 1$ for all k.

This approach also provides a way to quantify the inflation, due to background selection, in the estimated effect of selection on the variants within a given SNP class.

References:

1 Weir, B. S. & Cockerham, C. C. Estimating F-statistics for the analysis of population strucure. *Society for the Study of Evolution* **38**, 13 (1984).

2 Cockerhan, B. S. W. a. C. C. Estimating F-statistics for the analysis of population strucure. *Society for the Study of Evolution* **38**, 1358-1370 (1984).

3 McVicker, G., Gordon, D., Davis, C. & Green, P. Widespread genomic signatures of natural selection in hominid evolution. *PLoS Genet* **5**, e1000471, doi:10.1371/journal.pgen.1000471 (2009).

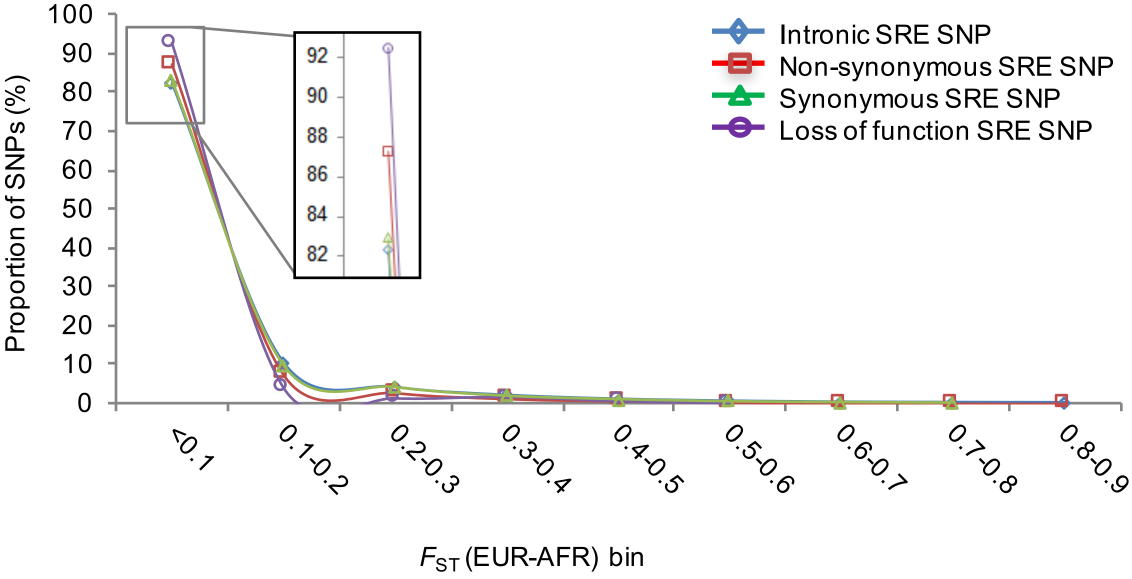

Supplementary figure 1. Global F_{ST} distribution of SRE SNPs in each SNP class for AFR-EUR comparison. X-axis is F_{ST} bin from the AFR-EUR comparison. Y-axis is the proportion of SRE SNPs in each F_{ST} bin. See Supplementary figure 2 for the ASN-EUR comparison and the AFR-ASN comparison

a.

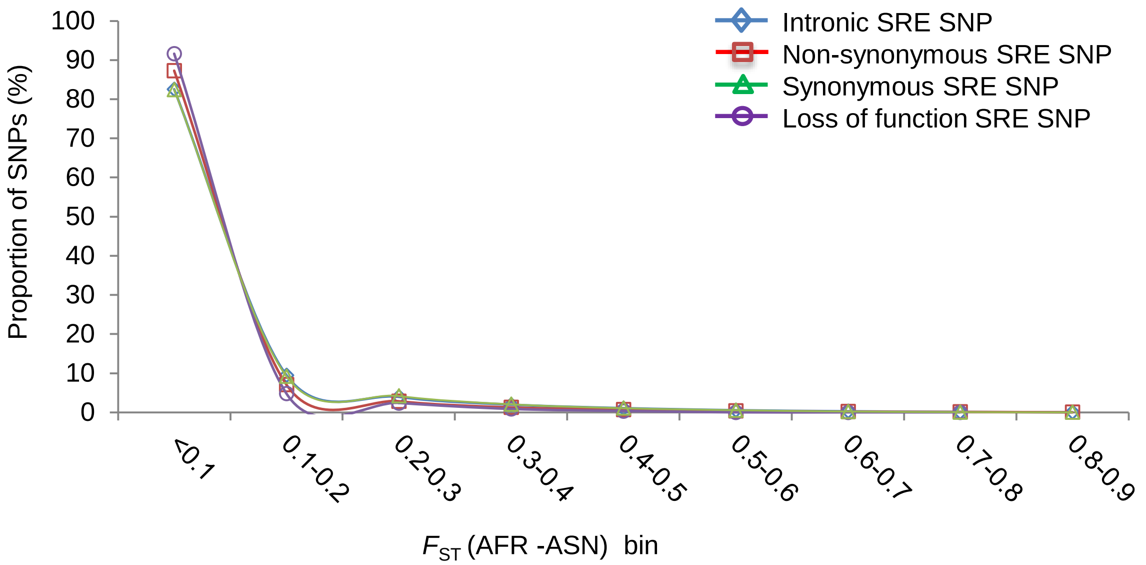

b.

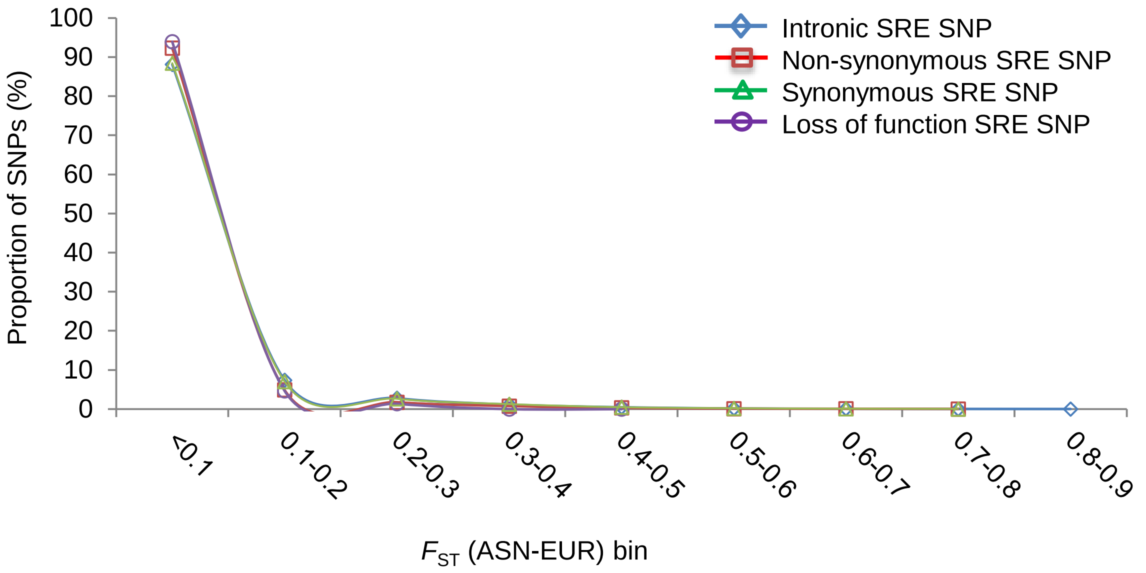

Supplementary figure 2. Global F_{ST} distribution of SRE SNPs in each SNP class. X-axis is F_{ST} bin and Y-axis is the proportion of SRE SNPs for each F_{ST} bin. (a) the AFR-ASN and (b) the ASN-EUR comparison.

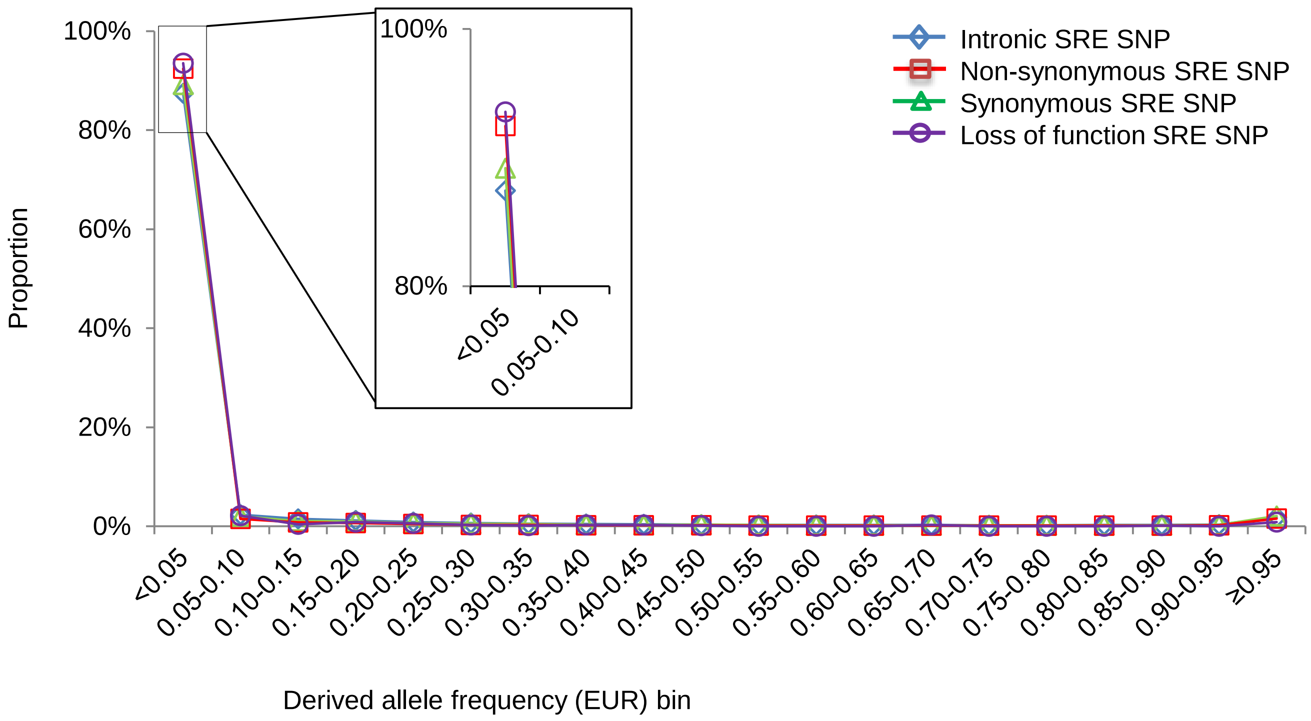

Supplementary figure 3. Proportion of derived alleles among the SRE SNPs with low F_{ST} (F_{ST} < 0.05). X-axis shows each DAF (EUR) bin and Y-axis is the DAF among SRE SNPs for the given DAF bin.

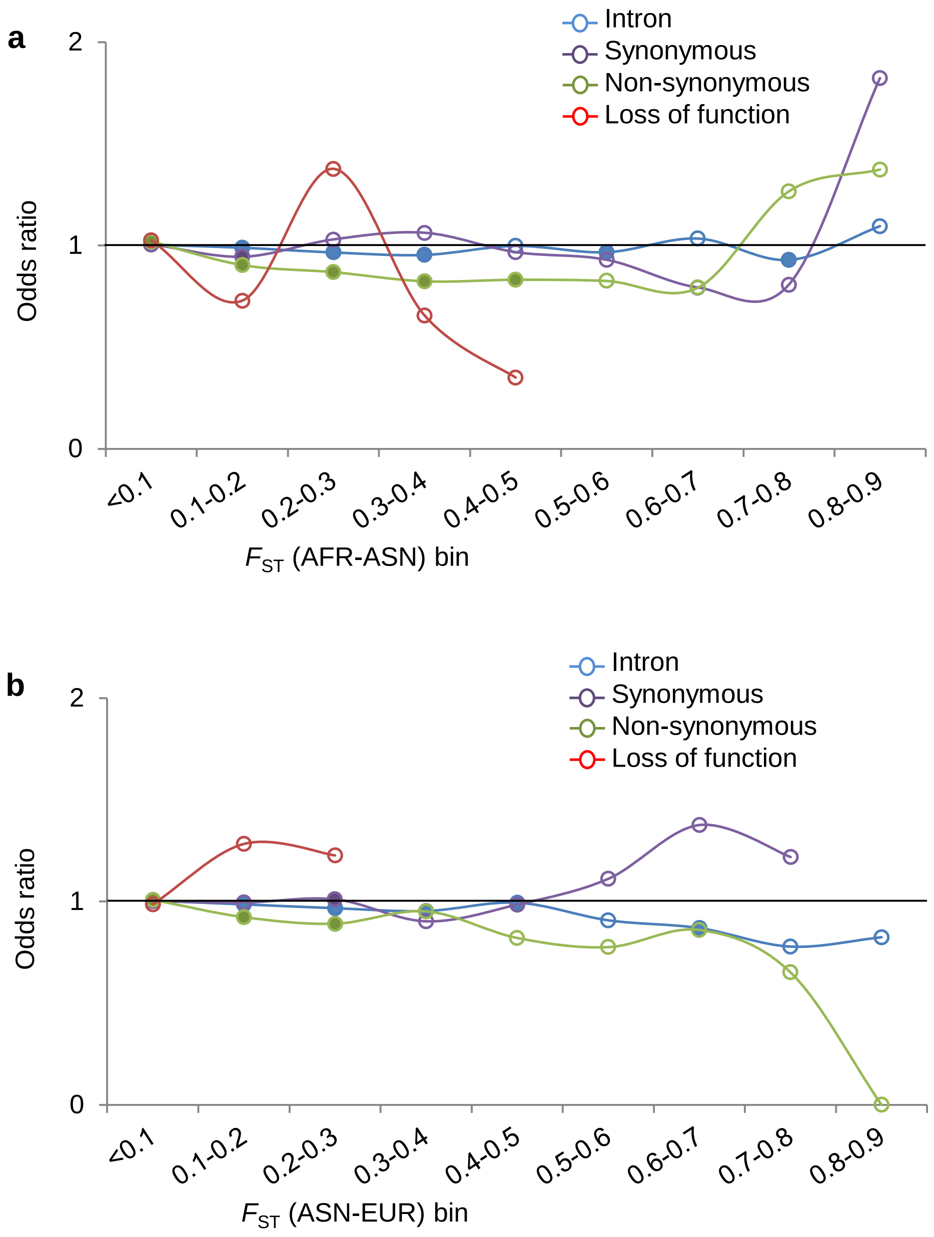

Supplementary figure 4. Relative proportion of SRE SNPs as a function of level of population differentiation (F_{ST}). X-axis shows each F_{ST} bin and Y-axis represents the odds ratio (OR) of SRE SNPs to non-SRE SNPs in each SNP class per F_{ST} bin (significance calculated using Fisher's exact test). Solid circle indicates statistical significance ($P < 0.05$). (a) AFR-ASN and (b) the ASN-EUR comparison.

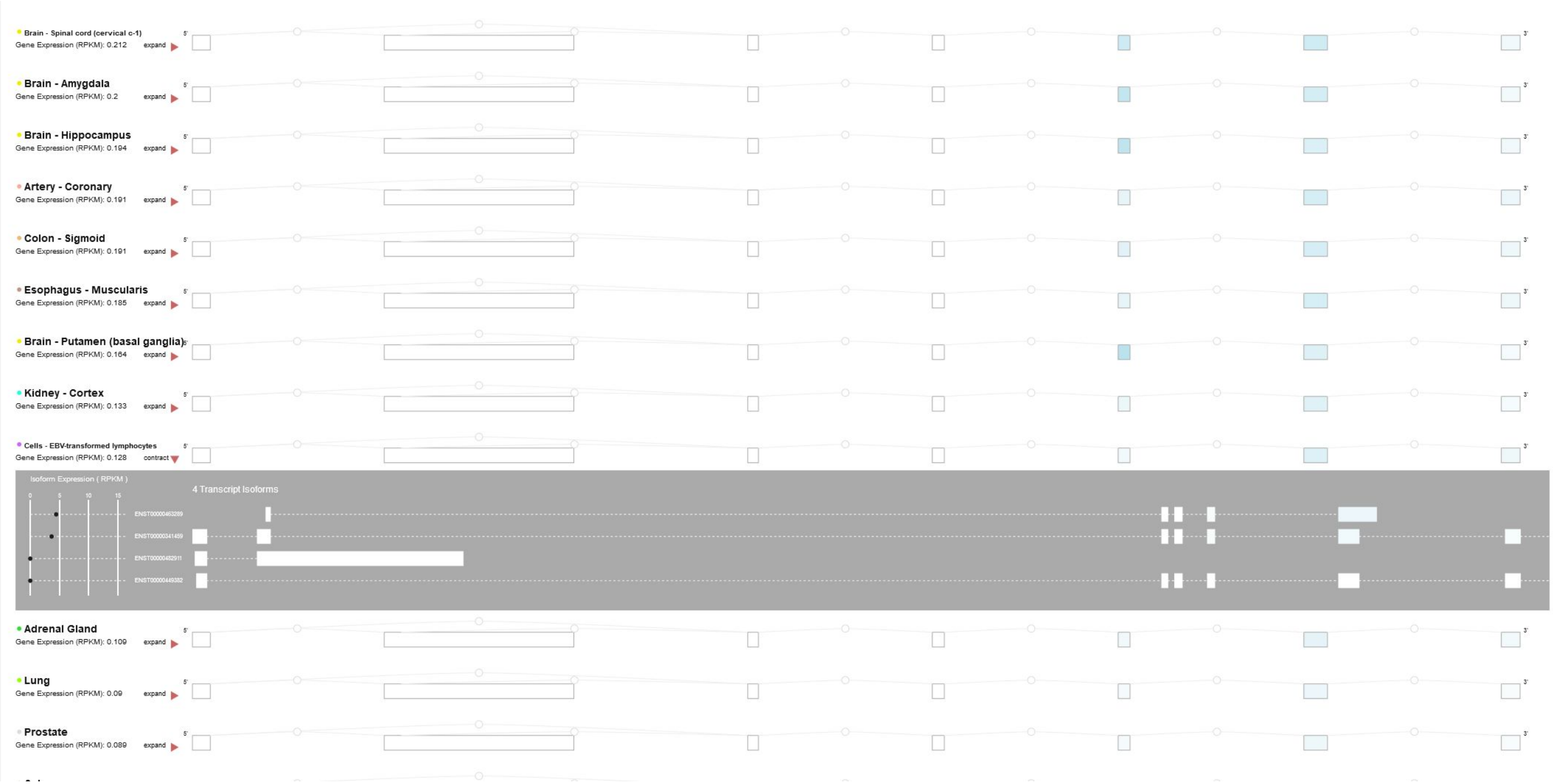

Supplementary figure 5. GTEx transcript view showing exon skipping of SLC24A5 in Skin-Sin Exposed (Lower Leg) tissues.

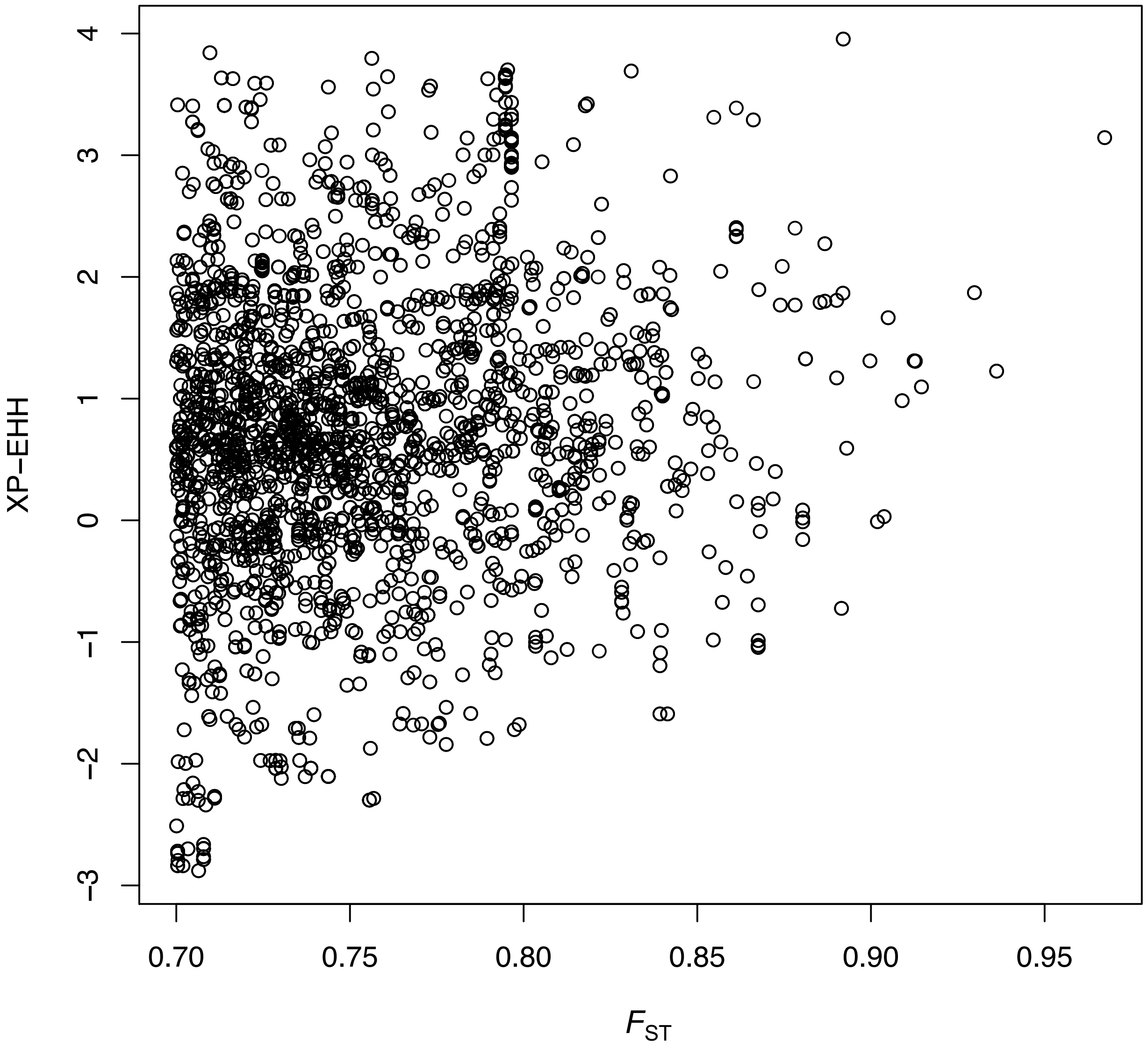

Supplementary figure 6. Correlation between F_{ST} and XP-EHH. For intronic SREs (ISE SNPs) with high F_{ST} (F_{ST} > 0.70), we found a significant spearman correlation (P < 2.2×10^{-16}, r = 0.11) between F_{ST} and XP-EHH and outlier SNPs for XP-EHH (with XP-EHH > 2 or XP-EHH < -2).

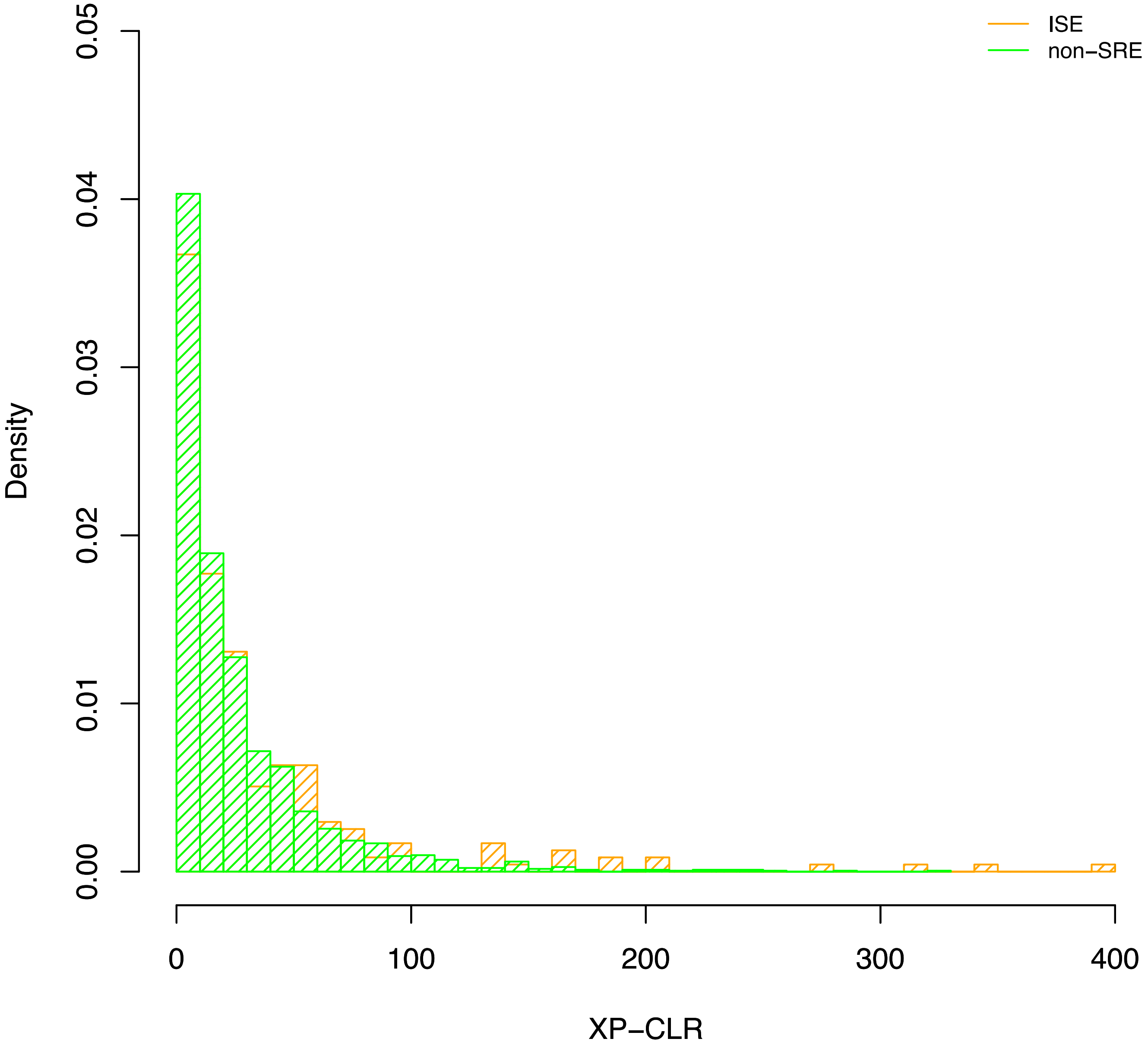

Supplementary figure 7. Distribution of XP-CLR for intronic SRE (ISE) SNPs and intronic non-SRE SNPs. The ISE SNPs showed a thicker tail-end XP-CLR distribution than non-SRE intronic SNPs across the genome.

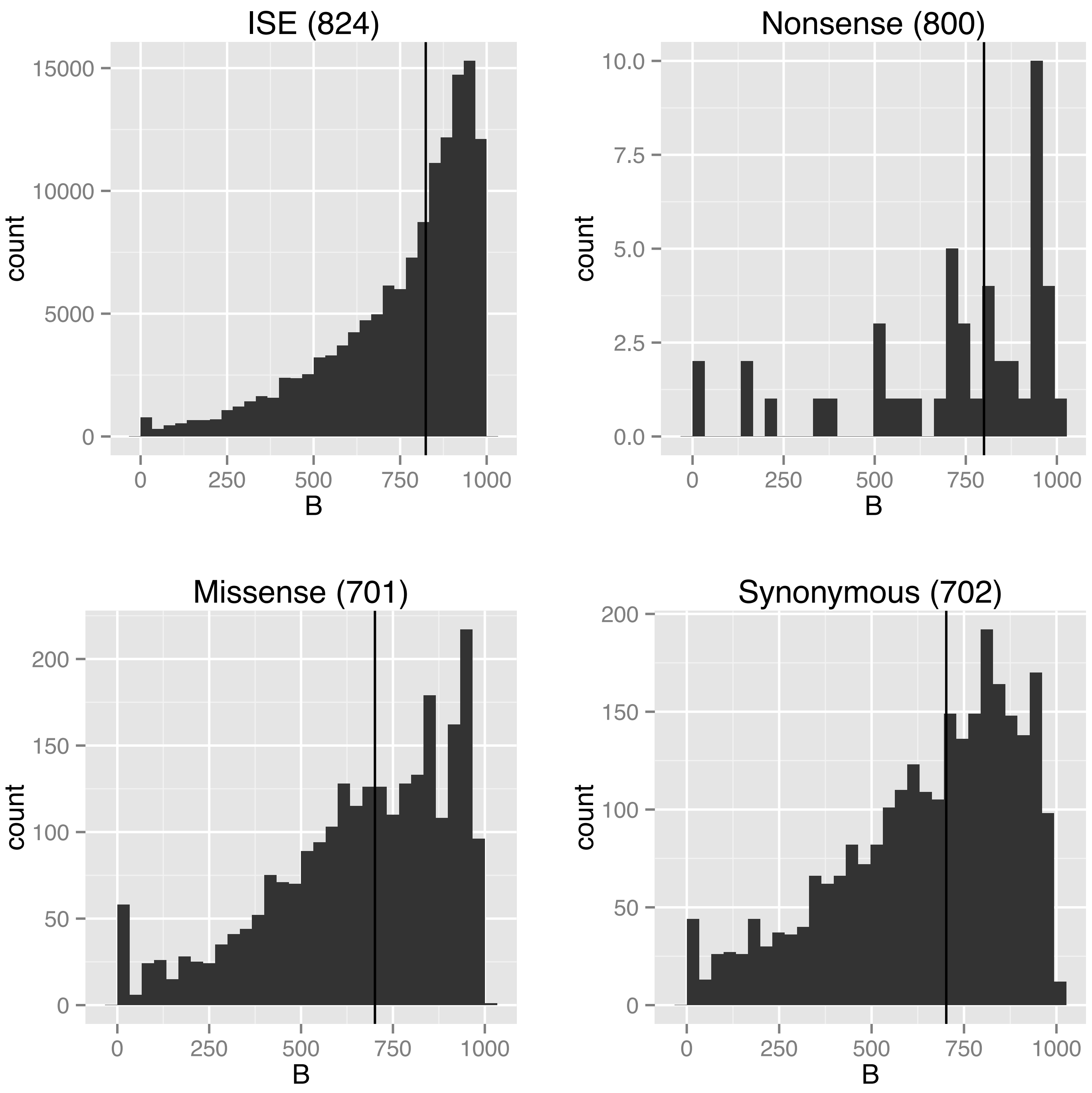

Supplementary figure 8. Patterns of background selection for SRE SNPs in each SNP class. The distribution of background selection for the SRE SNPs in each functional category is shown.

Supplementary Table 1. SRE SNPs and non-SRE SNPs by functional category.

	Intron	Synonymous	LOF	Non-synonymous
SRE SNP	2140482	119818	4675	175069
SRE SNP with exon skippin	2130021	71742	2438	103571
Non-SRE SNP	18026084	136870	7523	196079

Supplementary Table 2. Among the trQTLs with high F_{ST} (F_{ST} > 0.70), listed here are the transcripts with differential isoform usage (Mann-Whitney U test) between the European and African samples in the GEUVADIS dataset

Transcript ENST ID	P-value
ENST00000552361.1	7.29E-06
ENST00000519554.1	0.008549
ENST00000535045.1	0.009004
ENST00000337109.4	0.009802
